# Recent Progress in Polymer-Containing Soft Matters for Safe Mining of Coal

**DOI:** 10.3390/polym11101706

**Published:** 2019-10-17

**Authors:** Hetang Wang, Yunhe Du, Deming Wang, Botao Qin

**Affiliations:** 1 State Key Laboratory of Coal Resources and Safe Mining, China University of Mining and Technology, Xuzhou 221116, China; 2 Key Laboratory of Gas and Fire Control for Coal Mines (China University of Mining and Technology), Ministry of Education, Xuzhou 221116, China; dmwang@cumt.edu.cn (D.W.); btqin@cumt.edu.cn (B.Q.); 3 School of Safety Engineering, China University of Mining and Technology, Xuzhou 221116, China; 18361273012@163.com

**Keywords:** coal, safe mining, soft matter, polymer additive modification, polymer-based material

## Abstract

Safe mining is the premise and guarantee of sustainable development of coal energy. Due to the combination of excellent properties of polymers and traditional soft matters, polymer-containing soft matters are playing an increasingly important role in mine disaster and hazard control. To summarize the valuable work in recent years and provide reference and inspiration for researchers in this field, this paper reviewed the recent research progress in polymer-containing soft matters with respect to mine dust control, mine fire control, mine gas control and mine roadway support. From the perspective role of polymers in a material system, we classify mine polymer-containing soft matters into two categories. The first is polymer additive materials, in which polymers are used as additives to modify fluid-like soft matters, such as dust-reducing agents (surfactant solution) and dust-suppressing foams. The second is polymer-based materials, in which polymers are used as a main component to form high performance solid-like soft matters, such as fire prevention gels, foam gels, gas hole sealing material and resin anchorage agent. The preparation principle, properties and application of these soft matters are comprehensively reviewed. Furthermore, future research directions are also suggested.

## 1. Introduction

Coal is the most abundant and widely distributed fossil energy on earth. It plays a very important role in the worldwide energy supply and is an important support for global economic and social development [1]. For example, in England during the Industrial Revolution, coal became the main energy source to support vigorous development. From 1650 to 1850, per capita coal consumption in England increased 13-fold [2]. Over the past 20 years, coal has accounted for more than 25% of global energy consumption and more than 38% of global electricity comes from coal-fired power plants. Compared to about 48% of oil in the Middle East, 70% of natural gas in the Middle East and the Commonwealth of Independent States [3], the distribution of coal in the world is more disperse. The USA has the largest coal reserves in the world [4]. From 2008–2018, the USA produced 7407.5 Mt (million tons) of coal and consumed 5773.7 Mt of coal. For emerging economies, like China, India and South Africa, coal is the main fuel source for basic industries such as steel manufacturing, cement production and power development [5]. As a country with poor oil reserves, modest natural gas but rich in coal, China’s coal consumption accounts for about 50% of the world’s total [3]. China has made great efforts to reform and optimize its energy structure, the proportion of coal declined from 75% in 2010 to 69% in 2015 [6]. However, coal consumption continued to grow and still accounted for 58.2% in 2018 and thus the main energy status of coal has not changed. The demand for energy and the advancement of technology have prompted the vigorous development of the coal industry. Sustainability of coal production is an inevitable choice to maintain a stable world energy supply for many years in the future.

Safe coal mining is to the core of sustainable development of coal energy. With the increase in the depth, strength and scale of coal mining [7] and the development of comprehensive mechanization, problems such as mine dust, mine fires, mine gas and roof fall are becoming more significant. Large amounts of dust are generated during coal mining and transportation and dust concentrations at working sites significantly exceed the permissible values in many cases [8,9]. The dust concentration of a fully mechanized mining face can generally reach 2000 mg/m^3^; the dust concentration of a fully mechanized excavation face can reach 1000 mg/m^3^, whilst the respirable dust could account for about 20% of the total dust. According to statistics, nearly 28,000 new cases of pneumoconiosis were added in China in 2016, of which coal mine pneumoconiosis accounted for more than 50% [10]. Mine fires can produce toxic and harmful gases, threaten the lives and health of workers and pollute the environment, causing significant economic losses to enterprises [11]. Endogenous fire is the main form of mine fires, in which coal spontaneous combustion in the goaf of longwall faces occurs most frequently [12,13]. Uncontrolled coal fires cause annual coal losses of 10–20 million tones in China, equivalent to monetary losses of 125–250 million dollars [14]. Methane is the main component of mine gas. If the methane content in a coal seam is high or the methane concentration in the air is high, it will seriously threaten the safe production of an underground coal mine [15,16]. In 2012, a major gas explosion accident killed 45 people in Sichuan, China [17]. In the statistics of accidents in 2016, there were four large coal mine roof fall accidents in China, resulting in 40 casualties [18]. Such mine disasters seriously threaten the safety and the health of mine employees. Thus, controlling coal mine disaster/hazard, improving the mine environment and ensuring workers’ safety and health have become the priority for safety and sustainable development of the modern coal industry [19].

Polymer-containing soft matters have important value and broad application prospects in ensuring safe mining of coal. Soft matter refers to substances that are between solid and ideal fluids, including foam, gel and granular matter, for example. Polymers are macromolecule compounds, which are linked by covalent bonds by one or several structural units. The polymers give excellent performance in strength, hardness, heat resistance, corrosion resistance, abrasion resistance along with many other properties. Thus, they are widely used in packaging, plastics, construction, mining and other areas. Polymer materials have become one of the methods to improve the efficiency and safety of coal mine engineering [20]. A series of new, green and efficient polymer-containing soft matters are formed for improving the properties of conventional materials through physical or chemical methods. Such products of polymer-containing soft matters have made an outstanding contribution to dust control, fire prevention, gas drainage, roadway support and repair in coal mines. In general, there are two main directions of development: One is the polymer-surfactant system [21], which depends on the good viscosity, thermal stability and degradability of the polymers, such as environmentally-friendly and high-efficiency dust suppressants [22]. The other is the polymer-inorganic mixture system, which uses the good curing reaction and mechanical stability of the polymer [23], such as for coal and rock crushing reinforcement materials [24]. At present, the cost of dust-suppressing solution materials can be only $1.0–2.0 per kilogram. The characteristics of polymer-containing soft matters, such as safety, high efficiency and reasonable economy, are in line with the concept of modern coal mine safety and sustainable development.

In recent years, for the safe and green mining of coal, much research has been undertaken on polymer materials and technologies, with important progress made. However, few summaries have been presented in this regard. Hence, this paper will summarize and review the application and development of polymer-containing soft matters in coal mines in recent years. The aim of the paper is to comprehensively understand the development and application status of polymer-containing soft matters for safe mining of coalmines. In this paper, the main challenges faced by polymer-containing soft matters research in coal mining will be highlighted and suggestions for future development will also be given. This work could provide important reference and inspiration for the research and application of polymer material in mine safety.

## 2. Classification of Polymer-Containing Soft Matters in Coal Mine Safety

Soft matters are widely developed and applied in coal mines. For the safety and greening of coal mining, polymers are typically used as additives or basic materials to improve the original soft matter properties or synthesize new soft matters. These are especially applied in coal mine disaster/hazard control and are mainly divided into four categories: 1. Dust control: Dust suppressant (surfactant solution), dust suppression foam (aqueous foam, foam sol); 2. fire prevention and control: Suspended mortar, composite gel, three-phase foam and coatings slurry; 3. gas drainage: Gas hole sealing material; 4. roadway support and repair: Anchor net shotcrete, liquid accelerator, resin anchorage agent and roadway repair grouting material.

## 3. Polymer-Containing Soft Matters for Coal Mine Disaster/Hazard Control

### 3.1. Polymer Additive Soft Matters in Mine Dust Control

In relation to the prevention and control of mine dust, compared to ventilation and dust removal fans, the traditional water-spraying dust suppression, coal seam water injection and emerging foam dust suppression are more widely used [25,26,27,28,29]. The core concept is to improve and develop dust suppressant performance and researches on polymer-surfactant system have begun. Polymers have significantly changed the properties of solutions and foam [30]. Spray and water injection mainly reflect the properties of the solution liquid, while foam is the properties of gas–liquid two-phase, which leads to different research directions and development trends.

#### 3.1.1. Surfactant Solution

**Figure 1:**
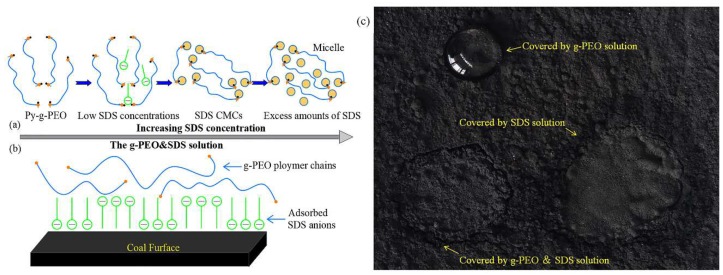
Principle of solution action and actual permeation: (**a**) Schematic overview of the industrial-grade polyethylene oxide (g-PEO) and sodium lauryl sulfate (SDS) solution; (**b**) schematic overview of dust control by the g-PEO&SDS solution; (**c**) penetration test of coal dust control in solutions containing SDS, g-PEO and g-PEO&SDS [32].

The effect of spray and water injection is related to the wettability and surface tension of the solution. The effect of dust control is related to the adhesion and consolidation ability of the solution. The better the wettability, the smaller the surface tension and thus the better the dust removal effect. The better the adhesion and consolidation performance, the less easy it is to raise dust. Most of the existing wettability studies are determined by contact angle or dust sinking experiments in solution. Dou [31] and others discussed the performance improvement of water-soluble polymer sodium carboxymethyl cellulose (CMC) and laboratory-made superabsorbent polymer (SA) on sodium dodecyl benzene sulfonate (SDBS) solution. The wettability was determined by the settling time of 0.2 g coal dust in 10 mL solution. While both of them reduced the surface tension of the solution, the effect of the absorbent polymer (SA) was more remarkable. It was pointed out that when the mass ratio of SA to SDBS was 1:4, the dust suppression effect was best. Based on the characteristics that the existing surfactants are easy to air dry and volatilize, Xi et al. [32] mixed the thermoplastic powder formed from the industrial-grade polyethylene oxide (g-PEO) (having a melting temperature close to the coal combustion temperatures) with sodium lauryl sulfate (SDS) to obtain a dust suppressant. The coal dust sinking test was used, although the wettability of the g-PEO&SDS solution was weaker than that of the SDS solution, the surface tension was larger, according to Equation (1)

(1) $$F_{c}=2\pi r\gamma cos\theta$$

where $F_{c}$ is the capillary adhesion, dyn, r is the particle radius, cm, γ is the suppressant surface tension, dyn cm^−1^ and θ is the contact angle, °. This indicates that the adhesion of g-PEO&SDS solution to particles was enhanced by the larger surface tension and as shown in Figure 1, the solidified layer can be formed on the surface of coal dust to reduce the evaporation of moisture in coal dust.

For the control of deposited dust in coal mine, Lai et al. [33] showed that the polymers could effectively reduce the dispersion of dust. Moreover, they indicated that microwaves could effectively improve the reaction rate of organic synthesis. Therefore, under 300 W microwave irradiation for two minutes, acrylic acid (AA), acrylamide (AAM) and oxidized starch can be polymerized to synthesize polymer dust suppressant (PSD). Then 10 mL PDS solution (2.0 g/L) was mixed with 6 g pulverized coal to prepare samples to determine their compressive strength, wind corrosion resistance and water corrosion resistance. The results show that the compressive strength reaches 0.293 MPa, the PDS solution remains stable at −12–50 °C. The compressive strength of coal samples soaked in water for many times remains unchanged and the loss rate of coal is only 1.2% after 3 h of strong wind erosion.

**Figure 2:**
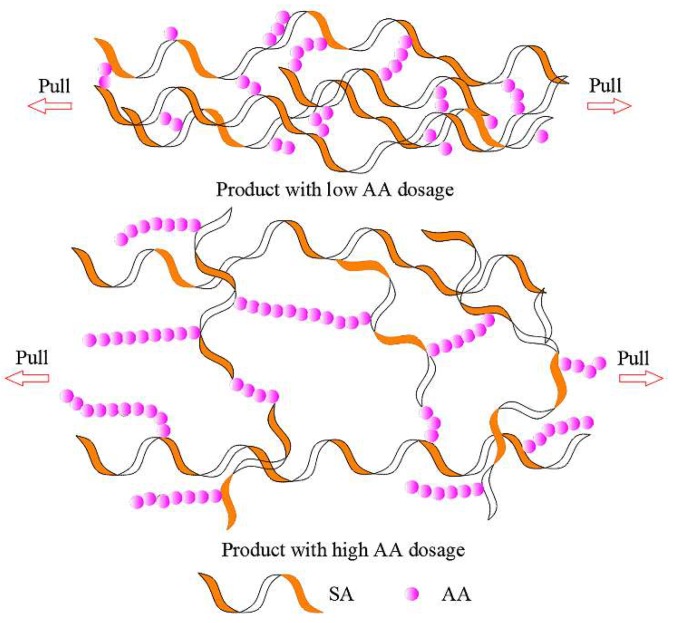
Microcosmic model of product in tension test [34].

**Figure 3:**
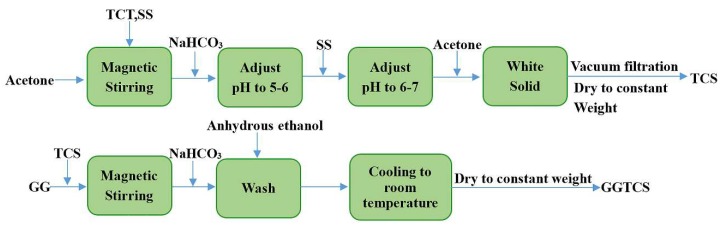
GGTCS synthesis flow chart (modified guar gum (GG) with cyanuric chloride (TCT) and sodium sulfamate (SS) forms the modified product GGTCS) [35].

**Figure 4:**
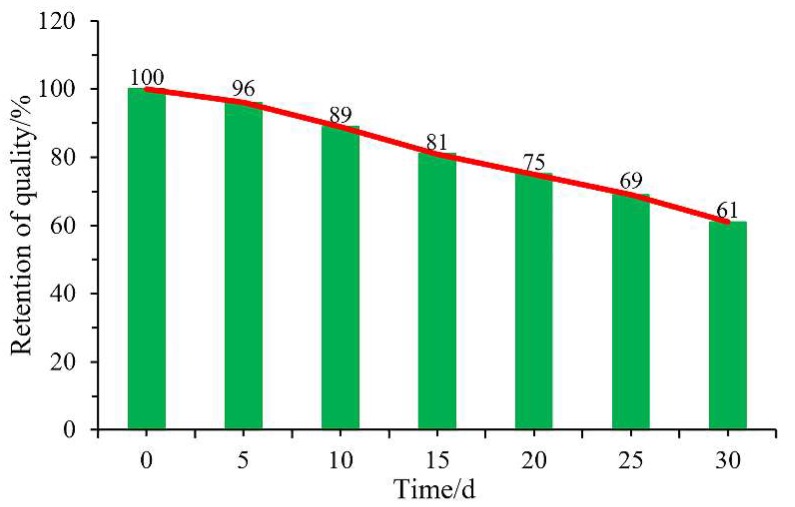
Degradability curve of dust suppressant film [35].

With the development of polymer-containing soft matters, some researchers have brought the concept of green environmental protection into the field of dust control. Zhou et al. [34] used sodium alginate as a matrix, because of its excellent hydrophilicity, viscidity, stability, low price, safety and non-toxicity, then chemically modified by graft copolymerization technology to synthesize an environmentally friendly dust suppressant under certain conditions. Caprolactam (CPL) and acrylic acid (AA) were selected as graft monomers. The results of a morphology test, contact angle analysis, sinking test, viscosity test and tensile test show that the new product had excellent viscosity, wettability, strength and deformation ability (Figure 2). The raw material is clean, the cost is low and the preparation is simple. As a natural, non-toxic and biodegradable polymer material, Zhang [35] modified guar gum (GG) with cyanuric chloride (TCT) and sodium sulfamate (SS) to form the modified product GGTCS (Figure 3). Thermogravimetric analysis showed that the thermal stability of the modified GGTCS increased from 268 °C to 285 °C. Using GGTCS as the main material, a new dust reducer of 0.8% GGTCS + 1.5% glycerol (GLY) + 0.1% sodium dodecyl benzene sulfonate (SDBS) + 0.02% fatty alcohol polyoxyethylene ether (AEO) was synthesized. It had good water retention and viscosity. When the coal dust was sprayed, the solidified film formed on the surface of the coal dust could bear up to 29 kPa of pressure. The solidified film can be buried in soil, with the quality reducing by 60% after one month (Figure 4). It also has good degradability characteristics.

**Figure 5:**
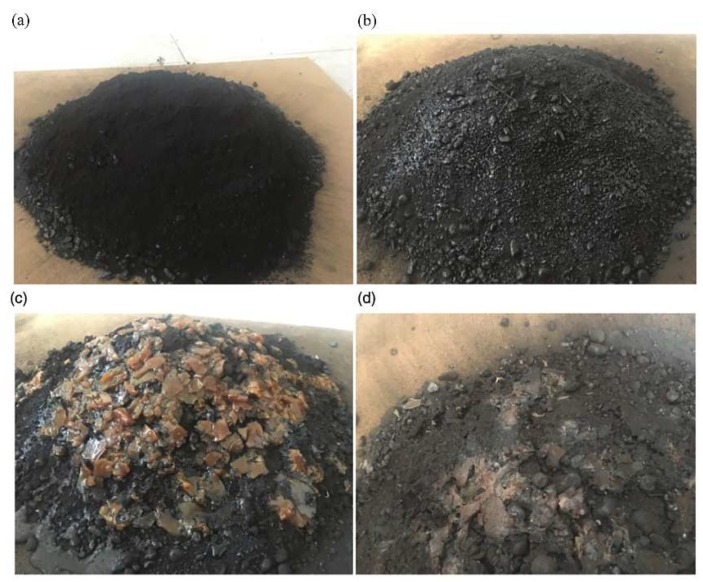
Pictures of the coal pile without and under different treatments: (**a**) Picture of the primary coal pile; (**b**) picture of the coal pile after being wetted; (**c**) picture of the coal pile after being coated with the coagulant; (**d**) picture of the coal pile after the application of coagulant [36].

For open pit mines, Zhou et al. [36] used sodium lignosulfonate as a surfactant and natural macromolecule to cross-link with acrylic acid to obtain cross-linking products and subsequently graft copolymerization with acrylamide to form a gel material. A new environmental dust suppressant was obtained for after crushing. Fourier transform infrared spectroscopy (FTIR), X-ray diffraction and differential scanning calorimetry (DSC) were used to determine the functional group characteristics, crystallization and thermal stability. Expansion kinetics, viscosity, film-forming hardness and peeling strength of spray solution were tested. The results show that it had better wind-proof characteristics, film-forming hardness and dust suppression ability (Figure 5).

#### 3.1.2. Aqueous Foam

**Figure 6:**
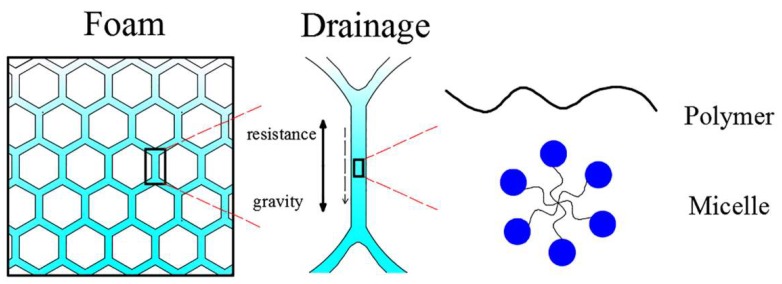
Foam drainage behavior of the surfactant solution with added polymer [37].

The efficiency of foam dust suppression is related to foam properties, which is characterized by stability, adhesion, wettability, initial liquid fraction and bubble size. Wang et al. [37] synthesized six test solutions by adding hydroxyethyl cellulose (HEC), polyacrylamide (HPAM) and polyvinyl alcohol (PVA) into two foaming agents, sodium alpha-olefin sulfonate (AOS) and polyoxyethylene octylphenol ether-10 (OP-10), respectively. The properties of the foam (wettability, surface viscosity and stability) were characterized by measuring the contact angle, viscosity modulus and foam discharge rate. It was indicated that adding polymers to AOS (OP-10) decreased the wettability of the foam, especially when more than 0.3 wt.‰. However, the polymers increased foam stability and foam surface viscosity. This was due to the two macromolecular chains, micelles and aggregates which existed in the liquid phase when the polymer dissolved in the surfactant solution, significantly increasing the viscosity of the liquid. The viscous force increased the strength of the foam liquid film, slowing down the foam discharge and increasing the stability of the foam (Figure 6). With the increase of polymer concentration, the effect was more obvious. Therefore, the low concentration of the polymer (0.1–0.3 wt.‰) has a better effect on the dust suppression ability of the foam.

Xu et al. [38] added polymer sodium carboxymethyl cellulose (CMC) to anionic surfactant fatty alcohol polyoxyethylene ether sulfate (AES) solution. In this instance it was shown that adding polymer can reduce foaming time at low gas flow rates and improve the initial liquid volume of the foam. Subsequently, Xu [39] and others studied the solution and foam properties of AES and partially hydrolyzed polyacrylamide (HPAM) at different concentrations. HPAM increases the surface tension of AES solution, but also increases its viscosity. Adding HPAM at low AES concentration could improve foamability and increase bubble size, because the increased bulk viscosity and the hydrogen bonds formed between AES molecule and HPAM chain stabilized the bubble film and reduced bubble damage. The increase of HPAM concentration can improve the liquid carrying capacity of foam and reduce the drainage rate. These findings indicate that adding water-soluble polymers to surfactant solutions can improve foam properties, thereby low concentration surfactants will be applied in coal mines.

**Figure 7:**
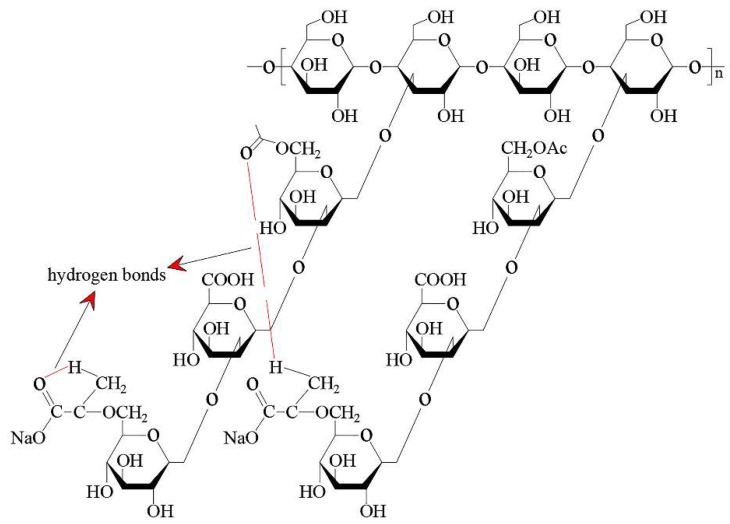
Structure of xanthan gum (XG) molecular chain [40].

**Figure 8:**
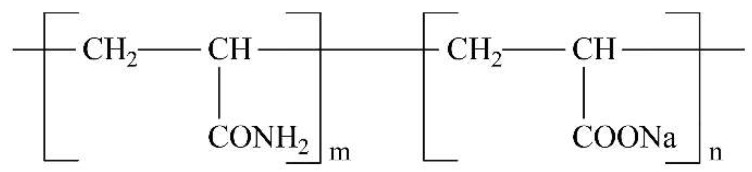
Structure of polyacrylamide (HPAM) molecular chain [40].

Wang [40] discussed the effect of xanthan gum (XG) and partially hydrolyzed polyacrylamide (HPAM) on the dust suppression foam produced by sodium dodecyl benzene sulfonat (SDBS) solution. The foam properties were characterized from three aspects: Foamability, liquid holdup and bubble size. The results showed that polymers improved foamability and liquid holding rate and the efficiency of XG was higher than that of HPAM. It is noted that polymer XG has many branched chains and hydrogen bonds in its structure. Hydrogen bonds can make XG curl and fold, so it has a large spatial effect (Figure 7). In addition, meshes and cavities can be formed to capture a large number of water molecules, thereby increasing the volume of liquid in the foam. HPAM is a typical linear water-soluble polymer (Figure 8). Its branches are far less complex than XG. Therefore, the foamabality, liquid holdup and bubble size of HPAM foam are weaker than those of XG.

#### 3.1.3. Foam Sol

**Figure 9:**
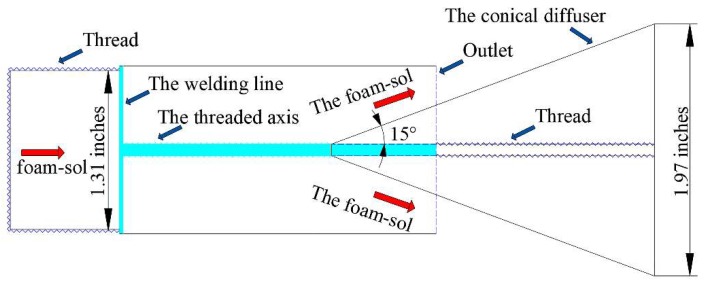
The detailed structural scheme of special conic nozzle used for spraying foam sol [41].

**Figure 10:**
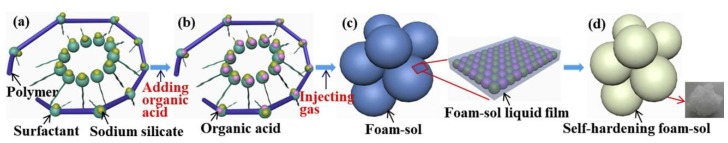
Chemical mechanism for the formation of self-hardening foam sol [43].

For the prevention and control of dust, Xi [41] put forward the foam sol with the function of dust catching, inhibition and isolation. Initially, foam sol consists of grease, acetate and byproducts generated in a slow crosslink reaction. A special conical nozzle was designed for spraying foam sol (Figure 9). Xi [42] also proposed a foam sol clay mixture, mainly composed of fly ash (FA), surfactant (n-pentylamine) and polyethylene oxide (PEO). When the mass concentrations were 200 g/L, 35 g/L and 1 g/L, the sol had the highest foam half-life, the best foaming performance and the highest stability. On the one hand, polyethylene oxide molecular chains are physically entangled to form hydrogen bonds or combine with water to form hydrogen bonds, which increases the viscosity of the solution. At the same time, polyethylene oxide can adsorb fly ash particles and reduce particle dispersion to obtain a stable sol solution. Furthermore, polyethylene oxide contains a large number of ether-oxygen units that can form associations with coal. Once the hydrophobic coal adsorbs polyethylene oxide aqueous solution, its hydrophobicity will change to hydrophilicity. The solution can effectively permeate the coal body and form an armor layer on the surface of the coal body after air-drying. Xi [43] then tried to synthesize a self-hardening foam sol, mainly composed of foaming solution (composed of 1 g/L PEO, 25 g/L sodium dodecyl sulfate 100 g/L sodium silicate and 874 g/L water) and organic acid. The formation mechanism of foam sol is shown in Figure 10. The results showed that when the organic acid concentration was 10–13 g/L, the foam sol had the best wetting ability, accepted loss of liquidity, self-hardening time and the material had good wind erosion resistance. For static dust suppression, the dust suppression rate could reach 100% at 12 m/s wind speed.

### 3.2. Polymer-Containing Soft Matters in Mine Fire Control

Fire from the spontaneous combustion of coal is one of the most common natural disasters in coal mines. It occurs when coal with spontaneous combustion tendencies encounters oxygen in the air, the heat generated by oxidation is greater than the heat lost to the surrounding environment and the heat accumulates, so that the coal temperature rises to the ignition point and ignites. The effective way to prevent spontaneous combustion of coal is to prevent coal from getting into contact with air [44]. In view of this problem, a variety of fireproof soft matters have been developed: Suspended mortar [45], composite gel [46], three-phase foam [47] and coating slurry [48] and on these bases, the high-quality fireproof polymer-containing soft matters have also been formed. Among them, suspended mortar and three-phase foam can be classified as polymer additive soft matters and composite gel and coating slurry belong to polymer-based soft matters.

#### 3.2.1. Suspended Mortar

Grouting, as the first means of fire prevention and control, is not only grouting yellow mud or fly ash, but also improving grouting materials according to the current situation and adapting to the requirements. Xu et al. [49,50] discussed the shortage of water resources in the west and north China and the poor effect of traditional grouting. A sand suspended mortar was developed and a composite additive was synthesized by mineral inorganic gel, organic polymer and dispersant. Inorganic gel can make water become viscous and expansible and colloid uniformly dispersed in water to form charged negative particles. Organic polymers are amylase polymers extracted from algae plants. They can combine with two valence ions such as Ca^2+^, Cu^2+^ and Pb^2+^ in inorganic gel to form a network space structure. The additive improves the quality of sand injection, reduces the need for water resources, increases the viscosity of grouting materials and produces a stable suspended mortar of sand. In the experiment, the suspended mortar of sand increased the Crossing-point temperature and activation energy of lignite and gas-fat coal (Table 1 and Table 2), delayed its oxidation reaction and effectively inhibited the spontaneous combustion of coal.

**Figure 11:**
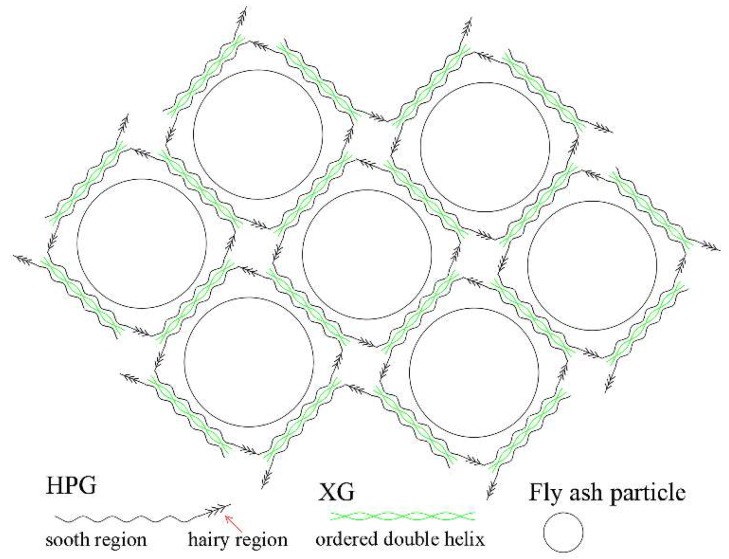
Schematic diagram of network structures between hydroxypropyl guar gum (HPG) and XG [51].

In order to improve the stability of fly ash mortar, Shi et al. [51] mixed the guar gum (GG) derivative hydroxypropyl guar gum (HPG) and xanthan gum (XG) at a mass ratio of 1:1 to obtain the mixture (BMS). Subsequently 300 g of fly ash (FA) particles were dispersed into 1 L of BMS solution with different concentrations and stirred to obtain the mixed mortar (FAS). The shear viscosities of BMS and FAS were measured by a rotational rheometer, the viscoelasticity was determined by dynamic angular frequency scanning, the stability was determined by a static sedimentation test, the fiber photographs of FAS samples were obtained using a scanning electron microscope and the flame retardant test was also carried out. The results showed that the viscosity of BMS solution and FAS mortar increased with the increase of HPG/XG concentration. This was due to the interaction between HPG and XG at a low shear rate, forming a gel network structure, supporting FA particles and preventing gravity settling, showing higher viscosity and excellent water retention (Figure 11). When the concentration of HPG/XG was more than 1.6 g/L, the yield stress of BMS exceeded the downward stress of the FA particles, which kept a stable state for 72 h and FAS mortar delayed the oxidation effect of coal more so than ordinary fly ash.

#### 3.2.2. Composite Gel

The inorganic gel has poor water holding capacity and thermal stability. Colloid composed of sodium silicate and ammonium salt is prone to produce toxic and harmful gases and the cost is high. For this purpose, the composite gels were developed by combining polymer and inorganic gel.

**Figure 12:**
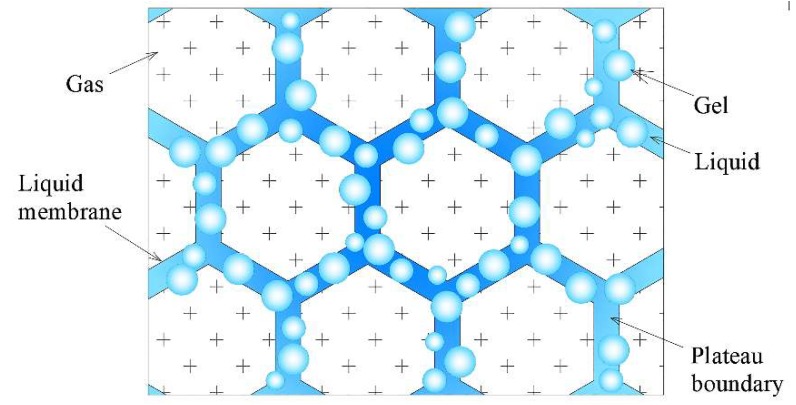
Schematic diagram of foam gel structure.

**Figure 13:**
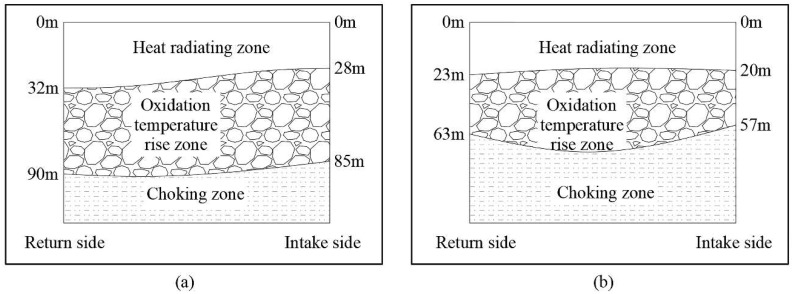
The variety of the three zones after injection of compound material into the goaf of the working face: (**a**) Before injection; (**b**) after injection [54].

Ren [52] introduced cationic polyacrylamide (CPAM), anionic polyacrylamide (HPAM) and carboxymethyl cellulose (CMC) into sodium silicate gel (WG) to obtain three kinds of polymer gels (CPAM/WG, HPAM/WG and CMC/WG). Subsequently, the crosslinking agent aluminum citrate was added to three kinds of polymer gels to obtain polymer-Al^3+^/WG gels. The compressive properties of the gels were analysed via a universal testing machine. The water-retaining property was tested in a weathering drying experiment. The microstructure was observed using a scanning electron microscope. The hydroxyl and methylene functional groups were analyzed by Fourier transform infrared spectroscopy. Micro EDS analysis was used to determine the elemental analysis. As shown in Figure 12, the results showed that the polymer/WG gels form a network gel system through intermolecular interaction and had good water retention ability. With the increase in polymer concentration, the water retention ability and strength of gels first increased and then decreased, this was related to the swelling degree of the polymers. When a crosslinking agent was added, a more stable network structure was formed with the polymer and the water retention ability became stronger. HPAM-Al^3+^/WG is an ideal fire extinguishing material because it has the highest water retention ability and causes significant inhibition on the oxidation of hydroxyl and methylene. For Hu et al. [53], the HPAM-aluminum citrate gel system was identified to be the most favourable gel system for fire suppression in underground coal mines. Huang [54] and others used sodium silicate as a gelling agent, ammonium salt and sodium salt as crosslinking agents, sodium bicarbonate as coagulant accelerator and non-toxic sodium polyacrylate (with strong water absorption) as a polymer additive to produce more environmentally-friendly gel material. Through field inspection (Figure 13), for the three zones of goaf, the heat dissipation zone was advanced by 8 meters, the oxidation temperature zone was shortened by 20 meters and the asphyxiation zone was advanced by 28 meters, showing good fire prevention and extinguishing performance.

Based on the principles of economy, environmental protection and high efficiency, environmentally-friendly gel materials have been investigated. Cheng et al. [55] grafted 2-acrylamide-2-methylpropane sulfonic acid (AMPS) and acrylic acid (AA) to form a polymer, mixed it with powdered corn straw to synthesize hydrogel and further, added the foaming agent and expandable graphite (EG) to produce an intelligent gel (IG) that can expand with the environment. The gel was compared with thermosensitive gel and polyacrylamide gel. It was shown to have good foaming properties, stronger thermal stability and provided a wider coverage of fire sources. The gel could effectively reduce heat radiation and CO production and expanded graphite after evaporation of water would made the gel expand again. Experiments showed that the gel had the shortest fire extinguishing time (Table 3). Hu et al. [56] synthesized self-gas foaming hydrogels using biopolymer chitosan (CS), attapulgite (APT), sodium acrylate (SA) as monomers and Na_2_CO_3_ as a foaming agent. Attapulgite (APT) is a type of chain-lamella clay mineral containing water and zeopan. It was shown that the swelling ratio (SR) decreased with the increase of CS. On the contrary, with the increase of APT, the SR of hydrogels increased initially and then decreased. The SR of hydrogel increased with the increase in foaming agent.

Qin [57] and others tried to apply other field materials to the suppression of spontaneous coal combustion. For instance, using super absorbent polymers made of polypropylene resin as physical inhibitor and ascorbic acid in the food industry as a chemical inhibitor, with a view to forming a new type of gel. When the oxidation temperature of coal is below 100 °C, physical inhibitor can absorb water from coal and form an inhibitory film on the surface of coal that prevents coal oxidation. Ascorbic acid dispersed in the three-dimensional structure of the polymer can be decomposed into hydrogen ions and reactive hydroxyl groups. The effect of physical inhibitor is weakened when the oxidation temperature exceeds 100 °C. Hydrogen ions decomposed from chemical inhibitor can react with alkoxyl and peroxide radicals produced by oxidation of aliphatic coal moieties to form hydroperoxide (R-OOH) and destroy free radicals to prevent further oxidation of aliphatic groups to CO or CO_2_. These hydroperoxides are then readily decomposed into alcohols and H_2_O. Simultaneously, the hydroxyl groups of the inhibitor react with the hydroxyl groups of the coal to form highly stable ether bonds, which deactivate the oxygen groups.

**Figure 14:**
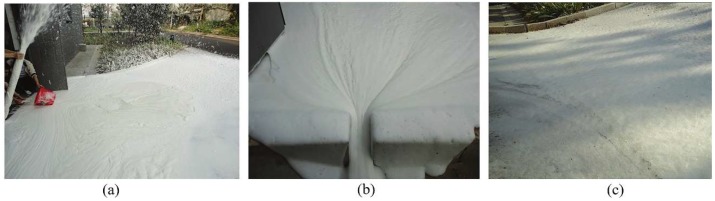
The foamed gel prepared in lab: (**a**) Foaming; (**b**) fluidity; (**c**) film forming ability [59].

Zhang [58,59] and others combined gel and foam to form a foam gel. The effects of thickeners and crosslinking agents on the foam gel were discussed. The gelation time was found to be reduced and the thermal stability improved as compared with conventional foam. Thickeners and crosslinking agents are polymers. Subsequently, the self-made foaming agent was mixed with the two polymers to form foam gel by air foaming. The apparent viscosity of the gel decreased with the increase in shear rate, which is consistent with the law of shear thinning of fluids. For high shear rates, thickeners and crosslinking agents do not occur cross-linking reactions in the foam film, which can satisfy the wide range of flow and diffusion in goaf. It was shown that 0.4% foaming agent, 0.3% thickener and 0.3% crosslinking agent could obtain a foam gel with high foaming ratio, strong stability and good fire resistance (Figure 14). Subsequently, at this concentration, the effects of the foaming ratio, addition of salts (NaCl, CaCl_2_ and AlCl_3_), pH value and temperature on the rheological properties of the foam gel were discussed [60]. The fire extinguishing time of thickening agent and crosslinking agent mixture at different concentrations was also examined [61].

#### 3.2.3. Three-Phase Foam

**Figure 15:**
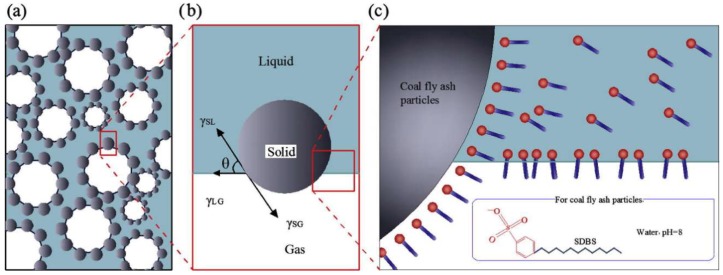
A structure model of three-phase foam to attach fly ash (FA) particles at the N2-water interfaces by tuning their surface-wetting properties: (**a**) Schematic illustration of the stabilization of gas bubbles with fly ash particles; (**b**) the adsorption of partially lyophobic FA particles at the gas–liquid interface, illustrating the balance in tension responsible for the attachment of particles; (**c**) a possible approach used to tune the wetting properties of originally hydrophilic particles by the absorbed sodium dodecyl benzene sulfonate (SDBS) molecules [63].

Three-phase foam consists of non-combustible solid matter (loess or fly ash), inert gas (nitrogen) and water and has good flowability. Compared with mortar and gel, the coverage of fire extinguishing is wider [62] and the fire-extinguishing methods are diverse. Qin and others [63] studied the effect of fly ash on the performance of three-phase foam. Figure 15 shows a three-phase foam structure model. It is suggested that the addition of a surfactant (SDBS) can promote the hydrophilic groups to be bound to the surface of fly ash and the hydrophobic tails enter into the water. This results in the transformation of hydrophilicity of fly ash to hydrophobicity and makes it easier to separate from water and form a three-phase foam stable structure. The addition of fly ash increases the retardation of water flow, slows down the drainage and makes the material exhibit excellent static (half-life of foam volume) and dynamic stability (viscosity changes with the shear rate). In addition, adding polymer carboxymethyl cellulose (CMC) can make the foam more stable.

**Figure 16:**
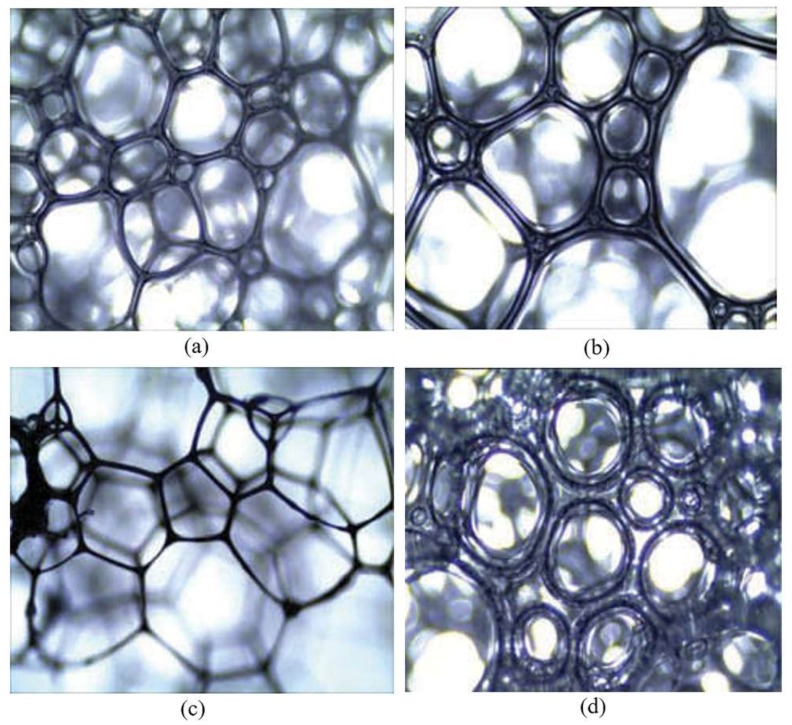
The microstructure maps of multi-phase foamed gel: (**a**) Without thickener and crosslinker; (**b**) only with thickener; (**c**) only with crosslinker; (**d**) both thickener and crosslinker [64].

Other researchers have considered the advantages of mortar, inert gas, gel and three-phase foam and developed comprehensive fireproofing soft matters. Zhang [64] and others synthesized a type of multiphase foam gel composite materials using fly ash, a foaming agent, a thickener, a crosslinking agent and nitrogen. The thickener is an anionic polysaccharide produced by the fermentation from xanthomonas campestris. The crosslinker is a galactomannan polysaccharide and belongs to polymers. The two reactions form a three-dimensional network structure and build a foam gel rigid skeleton, which has good water absorption, water retention and foam stability. As shown in Figure 16, the bubble distribution of polyphase foam gel without polymers or a single polymer is chaotic, the size dispersion is large; the difference between the maximum and minimum foam diameter is almost 10 fold and the foam liquid film is thinner. The addition of thickeners and crosslinking agents has a uniform size and the liquid film is thicker than before. When the foam bursts, the three-dimensional structure can still ensure the fly ash and slurry aggregate together form a cladding, with high strength and a certain degree of flexibility. It can compactly cover and pack coal, plug cracks and continuously and effectively prevent coal from absorbing oxygen, thus preventing coal oxidation.

#### 3.2.4. Coating Slurry

Compared with the suspension ability of mortar, the thermal stability, expansion property, water retention of the gel, the foamability, stability and fluidity of the three-phase foam, the coating material focuses on air tightness and mechanical properties. Phenol-formaldehyde foam [65] is widely used as an effective material for filling and blocking air leakages due to its heat resistance, flame retardance, good sealing and construction convenience. Hu [66] and others improved phenol-formaldehyde foam by using 1 mol phenol, 0.5 mol urea, 3 mol paraformaldehyde, 0.05 mol catalyst, for 3 h at 75 °C to synthesize phenol-urea-formaldehyde (PUF) composite foam sealing material. It was shown that PUF foam had high size stability, foaming ability and oxygen index. At the same time, polyethylene glycol (PEG) was used to improve the toughness and strength of the PUF foam [67]. PEG decreased the foam diameter, slightly decreased the thermal stability of the foam, but thickened the foam film. Subsequently, the effect of surfactants (silicone oil and Tween-80) on PUF foam was considered [68]. Hu [69] then optimized PUF foam formulation and compared to commonly used polymer foam (polyurethane foam, phenolic foam and urea formaldehyde foam). The composite foam had the greatest compressive strength, thermal stability, flame retardance and minimum foaming temperature and shrinkage rate, demonstrating excellent sealing performance.

**Figure 17:**
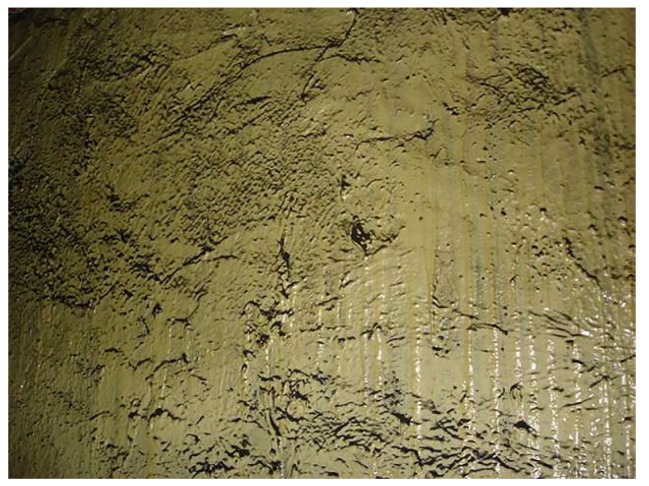
Practical application of modified polyurethane elastomer (PUE) in a coal mine [70].

Polyurethane is also an important polymer, with many airtight coating materials having been derived from it. Polyurethane elastomer (PUE) was first used as an air leakage sealant in roadways. Wu et al. [70] added surface modified TiO_2_ and SiO_2_ nanoparticles to obtain reinforced and toughened PUE nanocomposites. Surface modified nanoparticles were evenly dispersed in the PUE matrix, which improved its thermal stability and flame retardancy. The dual action of the dispersion system and hydrogen bond in PUE improves the mechanical properties of the material. The minimum elongation at break is more than 600%. The minimum tensile strength of the tested material is 2.45 MPa and the maximum is 3.21 MPa. The leakage rate decreased by 90%. Figure 17 illustrates a practical application diagram. Hu et al. [71] also obtained flame-retardant polyisocyanurate–polyurethane foam (PIR–PUR) by adding expandable graphite (EG) and dimethyl methyl phosphonate (DMMP).

**Figure 18:**
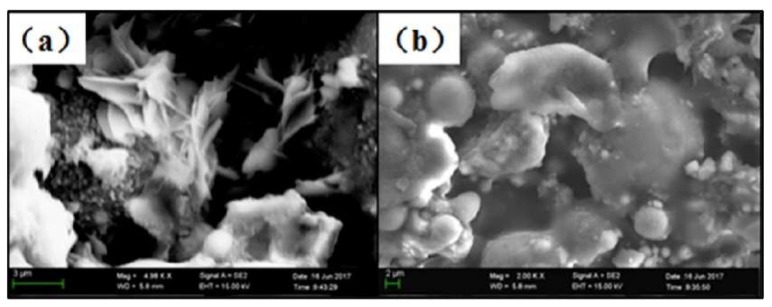
SEM image of fireproof material: (**a**) Hydrated product; (**b**) polymer film [74].

Past literature has also explored polymer emulsions other than polyurethane. Song et al. [72] first mixed styrene-acrylic emulsion with water and then added silica powder, Portland cement and flame retardant solid powder to synthesize a new sealing material. The results showed that the sealing material with 11 wt.% complex flame retardants (composed of aluminum hydroxide and chlorinated paraffin at a ratio of 3:8) had good performance when the retardants were. The tensile strength of this gas sealing material exceeds 2.25 MPa and the flame resistant property conformed to the safety standards of coal mines. Twardowska [73] has long shown that full mixing of fly ash and water has a higher air barrier effect than natural sealing materials. Su [74] first mixed cement and ethylene-vinyl acetate (EVA) copolymer emulsion with five kinds of industrial waste powder separately and then added water and defoamer to synthesize five kinds of fireproof materials. The five types of industrial wastes included fly ash (CFA), construction and demolition wastes (C&D waste), ferronickel slag (FNS), steel slag (SS) and red mud slag (RS). In the solution, EVA polymer is adsorbed on the surface of inorganic particles. As cement hydration gradually consumes interstitial water, the consolidation of polymer particles begins to form interlaced membranes, fill pores and cracks and wrap hydration products and aggregates [75]. This organic network ensures the compactness and overall microstructure of sealing materials. The permeability test and mechanical property test showed that the five materials have 3–4 orders of magnitude less permeability than the traditional materials and have good tensile strength. On the one hand, they depend on the high pozzolanic activity of the waste materials; On the other hand, they are affected by the film-forming properties of polymers (Figure 18). The air permeability coefficient, tensile strength and elongation at break of the best fly ash products were 4.17 × 10^−8^ m^2^/s, 2.14 MPa and 48.90%, respectively.

A. Tosun [76] showed that the polymer was a material with low oxygen permeability, thus the oxygen permeability of fiber reinforced polymer was studied and an epoxy resin/glass fiber composite with very low oxygen permeability and high mechanical resistance was developed. The cost of this material (80 g/m^2^) was $0.124–0.209.

### 3.3. Polymer-Based Soft Matters in Mine Gas Drainage

Gas explosion is one of the main disasters in coal mines, gas outburst has become more frequent in recent years [77]. Gas drainage by drilling is one of the most effective methods to prevent and control gas disasters and the efficiency of gas drainage largely depends on the sealing effect of the drillings. In the past, inorganic sealing materials mainly composed of cement mortar, cement fly ash, cement water glass and other cement-based soft matters were used. While the cement-based soft matters have a low cost and a wide range of sources, it is easy to precipitate during solidification and the volume shrinkage rate is typically more than 15% [78]. Moreover, the poor adhesion of cement-based materials can easily lead to the separation of pore walls and the formation of gas leakage channels [79], so their application is not ideal. In order to improve the sealing quality, polymer-containing sealing soft matters based on cement, fly ash and other basic raw materials have been extensively studied.

Xiang et al. [80] synthesized pore-sealing material with fly ash as the basic material, water-retaining agent, expanding agent, cellulose, resin, coupling agent and silicate. Compared with the traditional sealing materials (expansive cement, polyurethane and mucus), new material had good compactness, stability, fluidity and permeability. With the increase of water cement ratio, the stability of the material decreased, and the fluidity and permeability increased. Zhou et al. [81] produced a sealing material: The mass ratios of dispersant, expansive agent, polymer to cement were 1.2%, 0.1% and 6%, respectively. The ratio of water to cement was 1.5:1 in application. The results indicated that with the increase of polymer content, the flexural strength increased, the compressive strength decreased and the density increased after curing. This was due to the polymer being interwoven into a three-dimensional network structure, filled in to the slurry, enhancing the flexibility of the material, while permeating with cement to form a bridge, improving the material structure.

**Figure 19:**
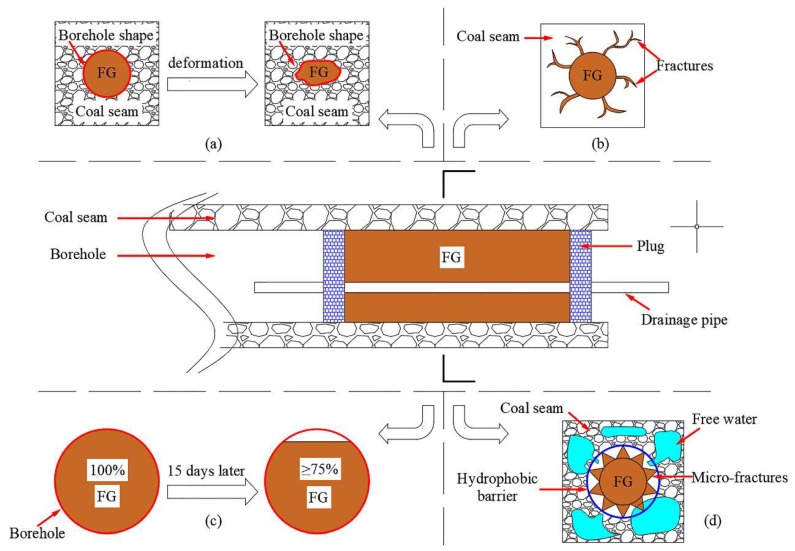
Sealing mechanism of the flexible gel (FG) for a borehole: (**a**) Borehole deformation adaptability; (**b**) high permeability; (**c**) preferable water retention; (**d**) hydrophobicity [83].

Polyurethane is the primary organic sealing material in gas sealing due to its fast curing speed, large expansion rate and excellent adhesion. Therefore, polyurethane-containing soft matters are also of consideration. Zhai [82] and others used cement, expansion agent, polyurethane resin, fibrin and coupling agent to synthesize a sealing material. However, the expanded polyurethane formed a cavity array, the diameter of the cavity was 700–1000 μm and there were pore space connections between the cavities. The overall air tightness of the sealing material was unsatisfactory. Subsequently, Zhai [83] combined polyurethane and fly ash to develop a flexible gel (FG) suitable for borehole deformation and excellent sealing performance. The basic components were fly ash, water retention agent, bulking agent, cellulose, resin, coupling agent and silicate. When the mixed materials began to solidify, the bulking agent fills the gaps between fly ash particles. Cellulose has the ability to retain moisture, bind to the borehole wall and increase the viscosity. The coupling agent was a type of plastic additive, which could improve the interfacial properties between synthetic resin and inorganic filler. The reactive epoxy groups in molecular structure of resin contains can form a three-dimensional polymer mesh structure. The silicate can produce a branched silicone, long-chain, mesh structure in the gel system, which has the synergies for increasing the gel strength. The material had strong adaptability to borehole deformation, good sealing and good water retention (Figure 19). It was found that the material and water mixed well in a low proportion and the sealing property was the best.

**Figure 20:**
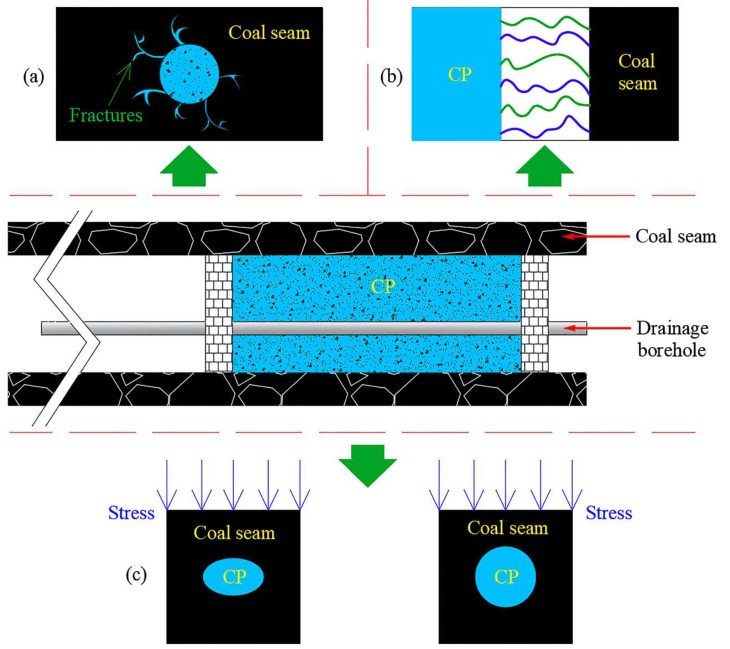
Sealing performance of composite polymer material (CP): (**a**) High fluidity and super sealing ability; (**b**) good combination with coal mass; (**c**) adapt to the deformation of borehole [85].

As far as polyurethane is concerned, it is not easy to diffuse around boreholes due to its high viscosity. The high-pressure injection results in the excessive permeation of mucus into the pores, which leads to a higher cost. Yang et al. [84] used clay, water retention agent, expansion agent and cellulose to obtain sealing material. Compared with cement mortar, it had superior expansibility and good water retention ability within 30 h. Li et al. [85] used coal dust as a filler, amino resin as a binder and other additives to synthesize a composite polymer material (CP). Amino resin is a linear polymer, which connects coal particles to play a role in stress transfer. Active groups such as carbonyl, amino and subamino groups on resin molecules have strong binding properties, which can enhance the cohesion between coal particles and the resin matrix. In addition, resin molecules contain many hydrophilic groups, such as ether bonds and hydroxyl groups, which can be combined with water molecules to form a stable dispersion system. –CH_2_OH, –NH– and other active groups in the resin molecule allow the polycondensation reaction to form a three-dimensional network structure in the presence of crosslinking agents. Therefore, coal dust particles can be encapsulated tightly in the resin matrix with specific mechanical strength. The toughening agent in the additives is also a linear polymer, which reacts with the resin molecule under acidic conditions and is embedded in the macromolecular chain of the resin to enhance the toughness and ageing resistance of the material. The product met the requirements of low pressure grouting and the pulverized coal particles could fill the holes and macro cracks, having a good sealing effect. Furthermore, the material had strong compressive strength and plastic deformation characteristics and could adapt to borehole deformation to a certain extent (Figure 20).

Zhang et al. [86] also used coal dust as basic material and used amino resin as binder, combined with other additives to synthesize a new composite sealing material. Its maximum penetration radius was 36 mm and it could be closely combined with coal. At the same time, it could also pass through cracks of 10 μm aperture and combine with coal, having a good sealing effect. Ge et al. [87] used Portland cement, early strength water reducer and polypropylene fibers to synthesize new hydraulic fracturing sealing materials, in which polypropylene fibers were used to increase the density of slurry, enhance the crack resistance and impact resistance of materials. At the same time, the slurry viscosity was increased, the shrinkage was reduced and the sealing property was enhanced.

### 3.4. Polymer-Containing Soft Matters in Mine Roadway Support and Repair

Anchorage is one of the key technologies to ensure the safety of coal mine and is used to protect from mine roof falls. The application of polymer-containing soft matters in controlling and repairing surrounding rocks of roadway is increasing of late: Stabilizing weak areas of rock, soil and coal; stabilizing areas where cracks exist; filling cracks, pores, etc. [88]. Murphy investigated a shale broken roof, indicating that polyurethane injection in the roof can chemically bond with rock mass and strengthen the roof strength [89]. For materials used in roadway support and repair, anchor net shotcrete and liquid accelerator are classified as Polymer additive soft matters, resin anchorage agent and repair grouting material are classified as polymer-based soft matters.

#### 3.4.1. Anchor Net Shotcrete

**Figure 21:**
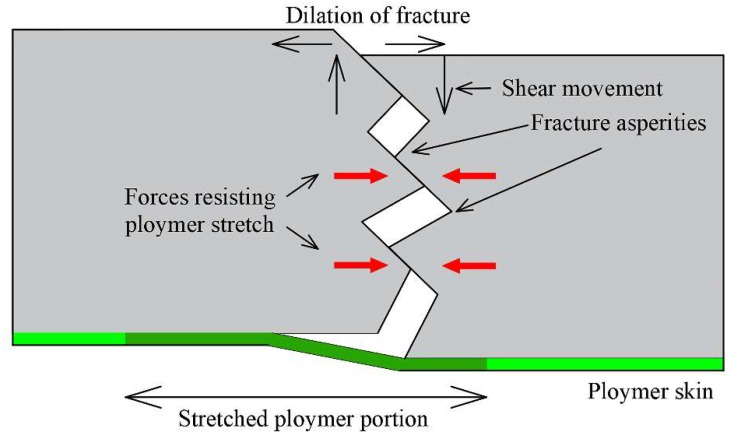
Shearing and dilation mechanisms of fracture surface [91].

For anchoring and shotcreting technology in a bolt-mesh support system, Australian researchers have been examining effective materials to replace anchor net support. Lukey et al. [90] indicated that the thin spray lining formed by spraying two commonly used material types (crosslinking polyurethane- or polyurea-based systems, and cement-reinforced water-dispersible systems based on ethylene-vinyl acetate copolymer) could provide auxiliary support for the anchor net. Meanwhile, Nemcik et al. [91] developed a powerful fiber reinforced polymer for spraying the top and surface of roadways, indicating that the material could be solidified in a few seconds, forming a polymer skin that could adhere well to the rock/coal surface and provide resistance to stratum displacement and fracture opening (Figure 21). In order to prove that the lining formed by fiberglass polymer is a suitable substitute for anchor net, Nemcik also tested the bearing capacity [92] and shear resistance [93] of the thin spray polymer lining in 2011 and 2013, respectively. The results showed that this kind of material had high compressive strength and displacement resistance, which was not inferior to the performance of anchor net and was better in some aspects. Shan et al. [94] also tested and compared the yield resistance of two kinds of fiberglass polymer commonly used as thin spraying lining and steel mesh to fully understand their failure modes in Australian mines.

**Figure 22:**
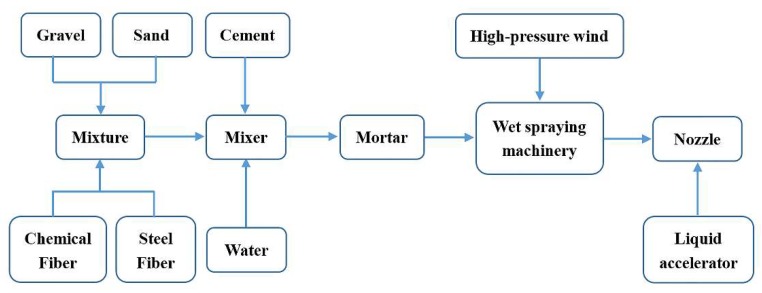
Wet shotcreting process of chemical fiber reinforced concrete.

Unlike Australia, in view of the complexity of China’s mines, the metal mesh bolt support is still commonly used in coal mines. Only in stratified mining, some mines will use high-strength polyester fiber flexible mesh as false roof instead of the metal mesh [95]. Wet shotcrete, as a synergistic anchor net support way, is very popular in Chinese mines. In order to improve the low compressive strength, weak ductility and easy cracking of concrete itself, Qi [96] and others discussed the application of inorganic fiber concrete in coal mine roadways, which was based on cement slurry, mortar or concrete and was synthesized by adding metal materials, inorganic fibers or reinforcing materials. Fiber reinforced polymer has been used in concrete due to its light weight, high strength, corrosion resistance and fatigue resistance. A series of cement-polymer composite soft matters have been developed [97]. Xu et al. [98] applied chemical fiber wet shotcrete (Figure 22) in mine field. Polypropylene fibers and steel fibers were added to concrete. The length of polypropylene fibers was 30 mm, the density was 0.91 g/cm^3^, the tensile strength was 500 MPa, the tensile elasticity modulus was 7 GPa, the elongation at break was 8% and the equivalent diameter was 0.6 mm. The results showed that chemical fibers increased the toughness and tensile strength of concrete to a certain extent. After three months of roadway excavation, there was no cracking of slurry skin and the wet spraying effect of concrete was significant.

#### 3.4.2. Liquid Accelerator

In order to accelerate the consolidation of wet shotcrete soft matters, research on liquid accelerators has also attracted much attention. Han et al. [99] developed a JL-1 low-alkali liquid accelerator, using sulphoaluminate and neutral sodium salts as the main accelerators. Polymer polyacrylamide was used to optimize the accelerator and improve its cohesiveness. The effect of the accelerator showed that the initial setting time was 2–4 min and the final setting time was 6–10 min. The strength of 28 d concrete does not decrease but increases slightly. Zhou [100] and others also developed a liquid accelerator consisting of 55% aluminium sulfate octadecahydrate, 4% sodium fluoride, 2.5% triethanolamine, 0.5% polyacrylamide, 5% bentonite and 33% water. Polymer polyacrylamide was also used as an additive. The good bonding properties of the polymer improved the cohesion of cement slurry and locks water, helping the solidification components shorten the solidification time.

#### 3.4.3. Resin Anchorage Agent

Bolt support is another important support method. Resin anchorage agent is widely used in bolt support [101]. Every year, resin anchorage is used in approximately 100 million rock anchors installed in mines in the United States [102]. Among them, resin materials are mainly unsaturated polyester resin, epoxy resin and polyurethane resin. Unsaturated polyester resin (UPR) anchorage material is a bonding material which is made up of UPR, catalyst and marble powder in a specific proportion. Han et al. [103] subsequently added steel sand and steel balls into anchorage material. After the pull-out test, it was shown that the size and quantity of steel particles can improve the anchorage performance of anchorage material to a certain extent. Su et al. [104] obtained the unsaturated polyester resin (UPR) anchorage material by mixing UPR and CaCO_3_ powder in a ratio of 5:1 and adding a suitable amount of accelerator. The thermal stability of UPR anchorage material in high temperature environments facing deep mining was studied. A study by Wang [105] examining the difficulty of supporting unsaturated polyester resin under water-contained conditions, synthesized an effective water-resistant anchorage agent by modifying polyester to contain hydrophobic groups and prevent water from intruding into the anchorage reaction system. Contrafatto et al. [106] studied the application effect of epoxy resin anchorage materials in natural stone (basalt, sandstone, limestone) and determined the minimum embedding depth of anchorage. Compared with traditional concrete anchorage material, the epoxy resin anchorage force was higher for basalt and limestone, but lower for sandstone.

#### 3.4.4. Roadway Repair Grouting

Grouting is one of the most important technologies for roadway maintenance and crack repair [107]. Early grouting soft matters were cement, silica sol, water glass, etc. Subsequently, some researchers tried to add a small amount of polymer into these materials. For example, Li used Portland cement, fly ash and 5 wt.% redispersible polymer to form emulsion slurry, then mixed with the aqueous foam formed by twelve alkyl sulfate and gelatin and added additives to form an inorganic polymer curing foam. The foam characteristics (stability, expansion rate) and mechanical properties (elastic compressive strength, elastic modulus, crushing engineering stress, plateau engineering stress and dense strain) were discussed [108,109].

**Figure 23:**
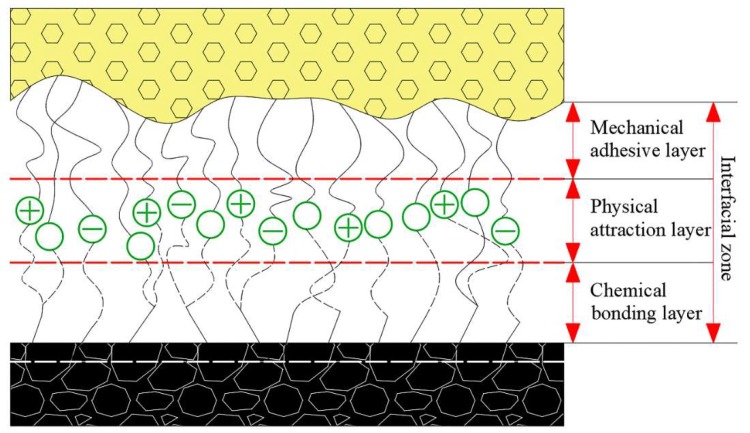
Interface model schematic diagram of polymeric foaming (PF) material [111].

In essence, because inorganic materials such as cement are easy to shrink, have poor permeability and short grouting length, organic polymer grouting soft matters such as polyurethane, epoxy resin, phenolic resin have emerged. Hu et al. [110] synthesized six phenolic foaming resins with different molar ratios of formaldehyde to phenol (F/P = 1.2, 1.4, 1.6, 1.8, 2.0, 2.2, 2.4). The results showed that with the increase of ratio, the trimer and tetramer of the resins gradually increased, the crosslinking degree of the phenolic resins increased and the chemical structure complexity of the polymers increased to form a three-dimensional structure. These resulted in an increase in the viscosity of the resin. While the foaming rate decreased, the foam density, compressive strength, thermal stability, oxygen index and flame retardancy increased. When the ratio reached 1.6, the size of the foam cells became more uniform. Zhang [111] and others used amino resin as a base material and added foaming agent, foam stabilizer, crosslinking agent, toughening agent and coupling agent to synthesize a polymeric foaming (PF) grouting material. Amino resin has many hydrophilic groups (–CONH_2_–, –NH_2_), which are easily bound to water molecules. Carbonyl (–C=O–), hydroxymethyl (–CH_2_OH) and imino (–NH–), which are active groups, have strong adhesion and can enhance the bonding strength of materials with the injected media. At the same time, the crosslinking agent in the material provides enough hydrogen ions to catalyze the condensation of resin molecules. As a result, the molecular weight of the resin is increased and the long molecular chain is changed into a network structure, which has a certain mechanical strength. Compared with superfine cement and aluminate cement, the slurry-coal interface formed by cement grouting is relatively loose. However, some chemical grafting reactions occur between PF and coal and a solid polymer coating is formed on the surface of coal body. The grouting reinforcement effect is improved (Figure 23).

**Figure 24:**
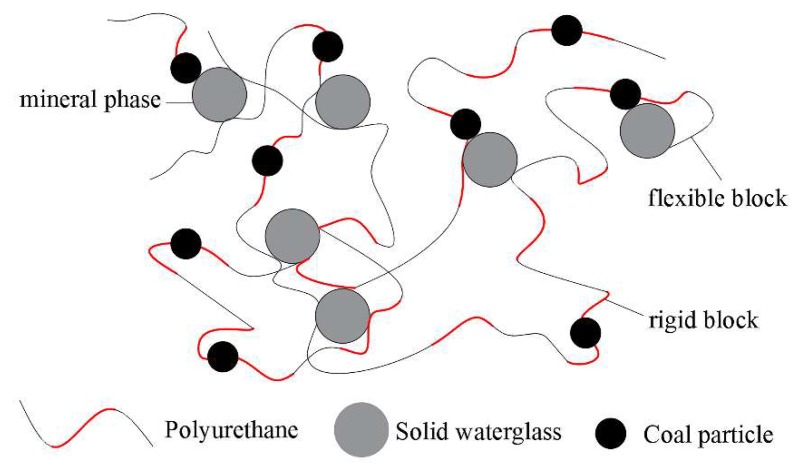
Bonding mechanism between sodium silicate/polyurethane adhesive and coal [112].

Due to the high cost of resin, organic and inorganic composite materials are typically produced. The excellent bonding property and high deformability of polyurethane (PU) and the low cost and high stability of sodium silicate (WG), means it is favorable to combine the two (PU/WG). Guan et al. [112] first added 50 g polyether polyol into 110.5 g sodium silicate and continued stirring until polyether polyols were completely dispersed to form emulsion. Then 60.5 g polymeric aromatic diphenylmethane diisocyanate (pMDI) was added. The polyurethane/sodium silicate adhesive solution was obtained by intense mixing. The compressive strength and flexural strength of coal/adhesives composition reached 21.4 MPa and 5.8 MPa, respectively. It is known that NCO groups in pMDI react with OH groups on coal surface first and then coal particles modified by NCO groups react with polyether polyols and sodium silicate, forming sodium silicate/polyurethane/coal compound. As shown in Figure 24, the obtained compound is composed of a polymer matrix and mineral phase. The structure of the polymer matrix is a combination of rigid and flexible blocks, coal particles are connected to polymer chains and solid sodium silicate is dispersed in the polymer network structure.

**Figure 25:**
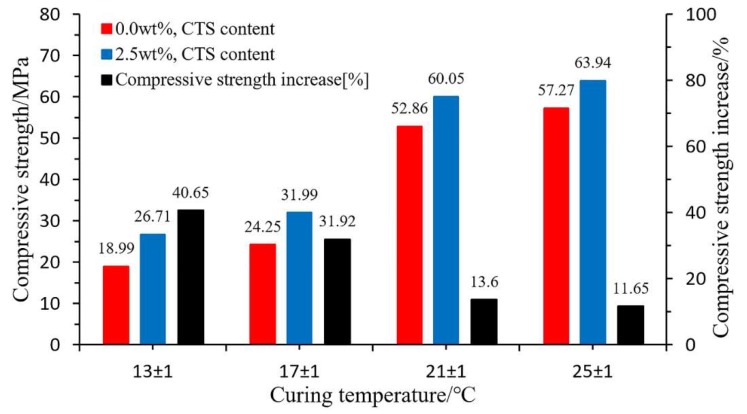
Compressive strength of the polyurethane (PU)/sodium silicate (WG)-0.0 wt.% and PU/WG-2.5 wt.% specimens at different curing temperatures for three days [113].

He et al. [113] indicated that polyurethane/sodium silicate (PU/WG) grouting soft matter was ideal for coal mine reinforcement. The compressive strength, fracture toughness, fracture energy, flexural strength and bending die of PU/WG grouting material were increased by 11.65–40.65%, 9.68%, 21.33%, 6.60% and 15.85%, respectively, by introducing 2.5 wt.% silane coupling agent 3-chloropropyltrimethoxysilane (CTS) (Figure 25). Zhang et al. [114] discussed the effects of dimethylbenzylamine (BDMA) and dibutyltin dilaurate (DBTDL) catalysts on the PU/WG grouting material. DBTDL accelerated the gel and curing reaction of the materials and BDMA increased the fluidity and compressive strength of the PU/WG. The synergistic catalysis of 0.1 wt.% BDMA and 0.1 wt.% DBTDL can not only improve the fluidity of the material but also reduce the curing time, with the compressive strength ultimately reaching 66.1 MPa.

A study by Gao et al. [115] examined the concept that chemical grouting materials such as polyurethane and urea resin are not suitable for mine environments with more water; thus, a new-type polymer grouting material was prepared with catalysts and vinyl epoxy resin, which was made from epoxy resin. This material had low viscosity, good polymerization reaction, accurate and controllable curing time and strong grouting ability. For mine aquifers and drift-sand layers, after solidification and expansion of material, resin and sand are bonded to form a compact structure, which has a better sand fixation effect and stronger bearing capacity (Table 4).

There are some safety production industry standards of the People’s Republic of China on coal mine polymer materials., such as AQ 1088-2011 (coal mine spraying polymer for sealing ventilation), AQ 1089-2011 (polymer material for consolidating coal and rock in mine) and AQ 1090-2011 (polymer foaming material for filling and sealing in coal mine). The polymer-containing soft matter discussed above generally meets or exceeds the requirements of relevant standards. For example, the tensile strength of PUE Nanocomposites meets the 2.0 MPa standard of AQ 1088-2011 (70). As another example, the minimum requirement for elongation at break is 30% and actual test of the material reaches 20 times the standard. Air tightness and compressive strength of the sealing material meet the standards of AQ 1090-2011 (81). Similarly, the compressive strength of PU/WG grouting material for coal and rock reinforcement exceeds the 60 MPa standard of AQ 1089-2011 (114).

## 4. Future Research Prospects

Regarding the development and application of polymer-containing soft matters in mines, researchers have used sophisticated experimental equipment and rigorous experimental methods to optimize material formulations and test material performance. Surface tension meter measured the tension of the solution. The liquid foam properties were measured by Foamscan. The shear viscosity characteristics of the flowing slurry were measured by rotational rheometer. The microscopic characteristics of the material were determined by a scanning electron microscope (SEM) and fourier transform infrared spectrometer and the mechanical properties of the solidified material were tested by a universal material testing machine. However, most of the materials are only tested by laboratory methods. For the research and application of engineering materials, more attention should be paid to the field effect. The combination of laboratory testing and field verification will better promote the development of polymer-containing soft matters in mines.

Overall, the study of polymer-containing soft matters is still in a period of rising development, presenting the research situation of far from adequate. It is reflected in that polymer-surfactant systems mainly focus on common surfactants (SDBS, SDS, AOS, AES, etc.) and polymers (HPAM, CMC, XG, etc.), while polymer-inorganic mixture systems mainly focus on common resin materials, especially polyurethane. Therefore, with the increasing demand for safe and green mining in coal mines and the progress of science and technology, the research of polymer-containing soft matters in mines has very broad prospects. The following are some suggestions on the future development directions.

As for the complexity of polymer and surfactant system, the main research directions in the future will expand from common materials to new surfactants and polymers. In the system, not only one kind of polymer is used, but also mixtures of many kinds of polymers are used and in this way, the performance of soft matters can be improved efficiently. Use of single polymer surfactants for research and development of mine materials would achieve the system optimization by two functions of a substance and reduce the cost of soft matters. Modifying polymers or synthesizing ideal polymers by popular graft copolymerization would achieve an economical and practical application mode. In addition, natural degradable green polymers will become the first choice of soft matters.

A polymer-inorganic mixture system is based on resin as the core, deepens the use of inexpensive inorganic materials such as fly ash and cement, improves the properties of soft matters and reduces the cost of materials. Facing situations of deep mining with high rock pressure, strong geothermal properties and strong water inrush, high-quality additives or multi-resin composites to enhance the chemical properties (cohesiveness, etc.) and mechanical properties (deformation resistance, etc.) of soft matters should be sought. Other resin polymers will gradually be introduced into the development of mine soft matters. At the same time, synthesizing super resin materials to replace existing materials will be required.

In addition, compared with linear polymers, bulk polymers are more likely to bond in the three-dimensional direction because of their large branched chains. It is believed that they will receive more attention and application in the research and development of polymer-containing soft matters in mines.

## 5. Conclusions

In this paper, the research progress of polymer-containing soft matters for safe coal mining is reviewed and the future development trends are suggested.

(1) According to the demand characteristics of the solid–liquid state of the soft matters, surfactant solution, aqueous foam, foam sol and three-phase foam are classified as fluid-like soft matters because of their strong liquidity. Suspended mortar, composite gel, coating slurry, gas hole sealing material, anchor net shotcrete and others are classified as solid-like soft matters that depend on the mechanical properties of materials after curing. Polymers are mainly used as additives or basic materials to improve the properties of the original soft matters or synthesize new soft matters, thus forming various high-quality soft matters in the application of different hazard prevention and control in coal mines.

(2) For different disaster prevention and control, polymer-containing soft matters have different emphases on property improvement. Surfactant solution has mainly a wetting, viscosity and film forming ability. Besides the above properties, dust suppression foam and foam sol also have good size characteristics and stability. The main matters of suspended mortar are fly ash, loess, sand and so on and mainly improve its cohesiveness and flow characteristics. The chemical gel has higher requirements for thermal stability, expansibility and water retention. A three-phase foam system is based on solid–liquid–gas, which requires foam to have good foaming properties, stability and viscosity. The main concerns of coating materials are mainly air tightness and mechanical properties (tensile strength, etc.). Gas sealing material is required to have good cohesion with the coal seam, permeability, expansibility and considerable deformation resistance. Anchor net shotcrete for roadway support should have certain toughness and deformation resistance (tension and compression resistance). Liquid accelerators need to reduce the consolidation time of the anchor–shotcrete materials. Resin anchorage materials should pay attention to water retention, cohesion with coal, shear and compression resistance. The repair grouting materials should have good permeability, fluidity and compressive strength.

(3) There are two aspects in the research of polymer-containing soft matters for mines. One is the polymer–surfactant system. This mainly depends on the three-dimensional structure formed by the polymer itself or when combined with other additives. This structure effectively wraps water, mud, fly ash, etc. The second is the polymer–inorganic mixture system used to achieve effective mixing of the polymers and inorganic substances (cement, mortar, fly ash). In addition to the three-dimensional structure of the polymer itself, it also depends on the chemical bonds formed by the polymer with coal and rock mass and the physical capillary adsorption of coal and rock pores, so that the materials have the ability to resist deformation.

(4) With regard to the development and application of polymer-containing soft matters, researchers have paid more attention to the principles of economy and green and environmental protection whilst aiming to maintain high-quality properties. Cheap and easily available inorganic materials and more environmentally friendly and efficient polymers tend to be used to obtain better polymer-containing soft matters.

## Figures and Tables

**Table 1:** Crossing-point temperature of coal samples.

Coal Samples	Crossing-Point Temperature (°C)				Ref
	Nothing Added	Soluble Glass	Calcium Chloride	Sand-Suspended Colloid	
Lignite	175.3	184.6	182.8	187.3	[50]
Fat-gas coal	166.3	182.7	180.5	178.2	

**Table 2:** Activation energy of coal samples.

Coal Samples	Activation Energy (kJ/mol)				Ref
	Nothing Added	Soluble Glass	Calcium Chloride	Sand-Suspended Colloid	
Lignite	77.00	92.09	74.48	95.37	[50]
Fat-gas coal	81.68	82.47	81.30	86.34	

**Table 3:** Fire-extinguishing times of the four materials.

Type of Materials	IG	TSG	PAM	Water	Ref
Time (S)	220	289	490	362	[55]

**Table 4:** Main properties of the cured material.

Category	Mechanical Characteristics	Ref
Density	1.6935 g/cm^3^	[115]
Compressive strength	10.38–13.09 MPa	
Strength of extension	0.32–0.53 MPa	
Elasticity modulus	401.37–490.64 MPa	
Poisson’s ratio	0.447–0.846	
Cohesion	0.866 MPa	
Internal friction angle	49.38°	

## References

[1] Wolde-Rufael Y. (2010). Coal consumption and economic growth revisited. Appl. Energy.

[2] Wrigley E.A. (2013). Energy and the English Industrial Revolution. Philos. Trans. R. Soc. Math. Phys. Eng. Sci..

[3] Dudley B. (2019). BP Statistical Review of World Energy.

[4] Bang G. (2010). Energy Security and Climate Change Concerns: Triggers for Energy Policy Change in the United States. Energy Policy.

[5] Yilmaz A.O., Uslu T. (2007). The Role of Coal in Energy Production-Consumption and Sustainable Development of Turkey. Energy Policy.

[6] Lin J., Fridley D., Lu H., Price L., Zhou N. (2018). Has coal use peaked in China: Near-term trends in China’s coal consumption. Energy Policy.

[7] Geng F., Saleh J.H. (2015). Challenging the Emerging Narrative: Critical Examination of Coalmining Safety in China, And Recommendations for Tackling Mining Hazards. Saf. Sci.

[8] Zhou W., Wang H., Wang D., Du Y., Zhang K., Kang W. (2019). The effect of coal proximate compositions on the characteristics of dust generation using a conical pick cutting system. Powder Technol..

[9] Brodny J., Tutak M. (2018). Exposure to Harmful Dusts on Fully Powered Longwall Coal Mines in Poland. Int. J. Environ. Res. Public Health.

[10] Wang T., Li Y., Zhu M., Yao W., Wu H., Ji X., Hu Z., Shen H., Fan X., Ni C. (2018). Association Analysis Identifies New Risk Loci for Coal Workers’ Pneumoconiosis in Han Chinese Men. Toxicol. Sci..

[11] Liang Y., Zhang J., Ren T., Wang Z., Song S. (2018). Application of ventilation simulation to spontaneous combustion control in underground coal mine: A case study from Bulianta colliery. Int. J. Min. Sci. Technol..

[12] Brodny J., Tutak M. The impact of the flow volume flow ventilation to the location of the special hazard spontaneous fire zone in goaf with caving of operating longwalls. Proceedings of the 16th International Multidisciplinary Scientific GeoConference: SGEM.

[13] Brodny J., Tutak M. Analysis of gases emitted into the atmosphere during an endogenous fire. Proceedings of the 16th International Multidisciplinary Scientific GeoConference: SGEM, Vienna GREEN Extended Scientific Sessions.

[14] Xi Z., Sun X. (2016). Effectiveness of Thermoplastic Powder to Retard Self-Heating and Spontaneous Combustion of Coal. Combust. Sci. Technol..

[15] Tutak M., Brodny J. (2017). Analysis of Influence of Goaf Sealing from Tailgate on the Methane Concentration at the Outlet from the Longwall. IOP Conf. Ser. Earth Environ. Sci..

[16] Tutak M., Brodny J. (2018). Analysis of the Impact of Auxiliary Ventilation Equipment on the Distribution and Concentration of Methane in the Tailgate. Energies.

[17] Meng X., Liu Q., Luo X., Zhou X. (2019). Risk assessment of the unsafe behaviours of humans in fatal gas explosion accidents in China’s underground coal mines. J. Clean. Prod..

[18] Wang X., Meng F. (2018). Statistical Analysis of Large Accidents in China’s Coal Mines in 2016. Nat. Hazards.

[19] Kong B., Li Z., Yang Y., Liu Z., Yan D. (2017). A Review on the Mechanism, Risk Evaluation, and Prevention of Coal Spontaneous Combustion in China. Environ. Sci. Pollut. Res..

[20] Chubrikov A. Use of Polymer Technologies in Kuzbass Coal Mining Industry. Proceedings of the 2007 International Forum on Strategic Technology.

[21] Krstonošić V., Milanović M., Dokić L. (2019). Application of Different Techniques in the Determination of Xanthan Gum-SDS and Xanthan Gum-Tween 80 Interaction. Food Hydrocoll..

[22] Jin H., Nie W., Zhang H., Liu Y., Bao Q., Wang H. (2019). Preparation and Characterization of a Novel Environmentally Friendly Coal Dust Suppressant. J. Appl. Polym. Sci..

[23] Singer G., Sinn G., Schwendtner K., Lichtenegger H.C., Wan-Wendner R. (2018). Time-Dependent Changes of Mechanical Properties of Polymer-Based Composite Materials for Adhesive Anchor Systems. Compos. Struct..

[24] Sun Z., Zhang J., Sun Y. (2019). Feasibility of A Polymer Foaming Agent as a Grouting Material for Broken Coal Masses. Adv. Civ. Eng..

[25] Wang H., Du Y., Wei X., He X. (2019). An Experimental Comparison of the Spray Performance of Typical Water-Based Dust Reduction Media. Powder Technol..

[26] Wang H., Wang D., Wang Q., Jia Z. (2014). Novel Approach for Suppressing Cutting Dust Using Foam on A Fully Mechanized Face with Hard Parting. J. Occup. Environ. Hyg..

[27] Wang H., Wei X., Du Y., Wang D. (2019). Experimental investigation on the dilatational interfacial rheology of dust-suppressing foam and its effect on foam performance. Process Saf. Environ. Prot..

[28] Wang H., Xuan W., Zhang Z., Qin B. (2019). Experimental investigation of the properties of dust suppressants after magnetic-field treatment and mechanism exploration. Powder Technol..

[29] Wang H., Chen X., Xie Y., Wei X., Liu W.V. (2019). Experimental study on improving performance of dust-suppression foam by magnetization. Colloids Surf. A Physicochem. Eng. Asp..

[30] Bureiko A., Trybala A., Kovalchuk N., Starov V. (2015). Current Applications of Foams Formed from Mixed Surfactant–Polymer Solutions. Adv. Colloid Interface Sci..

[31] Dou G., Xu C. (2017). Comparison of Effects of Sodium Carboxymethylcellulose and Superabsorbent Polymer on Coal Dust Wettability by Surfactants. J. Dispers. Sci. Technol..

[32] Xi Z., Feng Z., Li A. (2017). Synergistic Coal Dust Control Using Aqueous Solutions of Thermoplastic Powder and Anionic Surfactant. Colloids Surf. A Physicochem. Eng. Asp..

[33] Lai S., Chai Q., Wang B., Yang N. (2012). Preparation and Application of Polymer Dust Suppressants in Coal Transportation under Microwave Irradiation. Adv. Mater. Res..

[34] Zhou G., Ma Y., Fan T., Wang G. (2018). Preparation and Characteristics of a Multifunctional Dust Suppressant with Agglomeration and Wettability Performance Used in Coal Mine. Chem. Eng. Res. Des..

[35] Zhang H., Nie W., Wang H., Bao Q., Jin H., Liu Y. (2018). Preparation and Experimental Dust Suppression Performance Characterization of a Novel Guar Gum-Modification-Based Environmentally-Friendly Degradable Dust Suppressant. Powder Technol..

[36] Zhou G., Fan T., Xu M., Qiu H., Wang J., Qiu L. (2018). The Development and Characterization of a Novel Coagulant for Dust Suppression in Open-Cast Coal Mines. Adsorpt. Sci. Technol..

[37] Wang H., Wei X., Du Y., Wang D. (2019). Effect of Water-Soluble Polymers on the Performance of Dust-Suppression Foams: Wettability, Surface Viscosity and Stability. Colloids Surf. A Physicochem. Eng. Asp..

[38] Xu C., Wang D., Wang H., Zhang Y., Dou G., Wang Q. (2017). Influence of Gas Flow Rate and Sodium Carboxymethylcellulose on Foam Properties of Fatty Alcohol Sodium Polyoxyethylene Ether Sulfate Solution. J. Dispers. Sci. Technol..

[39] Xu C., Wang D., Wang H., Hu J., Zhu X., Zhang Y. (2018). Effect of Partially Hydrolyzed Polyacrylamide on The Solution and Foam Properties of Sodium Alcohol Ether Sulfate. Colloids Surf. A Physicochem. Eng. Asp..

[40] Wang Q., Wang D., Shen Y., Wang H., Xu C. (2017). Influence of Polymers on Dust-Related Foam Properties of Sodium Dodecyl Benzene Sulfonate with Foamscan. J. Dispers. Sci. Technol..

[41] Xi Z., Jiang M., Yang J., Tu X. (2014). Experimental Study on Advantages of Foam–Sol in Coal Dust Control. Process Saf. Environ. Prot..

[42] Xi Z., Jin L., Liew J.Y.R., Li D. (2018). Characteristics of Foam Sol Clay for Controlling Coal Dust. Powder Technol..

[43] Xi Z., Zhou S., Jin L. (2019). Experimental Investigation of Self-Hardening Foam-Sol for Controlling Diffusion of Static Coal Dust. Powder Technol..

[44] Song Z., Kuenzer C. (2014). Coal fires in China over the last decade: A comprehensive review. Int. J. Coal Geol..

[45] Colaizzi G.J. (2004). Prevention, Control and/or Extinguishment of Coal Seam Fires Using Cellular Grout. Int. J. Coal Geol..

[46] Deng J., Xiao Y., Lu J., Wen H., Jin Y. (2015). Application of Composite Fly Ash Gel to Extinguish Outcrop Coal Fires in China. Nat. Hazards.

[47] Zhou F., Ren W., Wang D., Song T., Li X., Zhang Y. (2006). Application of Three-Phase Foam to Fight an Extraordinarily Serious Coal Mine Fire. Int. J. Coal Geol..

[48] Hussain F., Saydam S., Mitra R., Cinar Y. (2012). Experimental Study for Reducing Gas Inflow by Use of the Thin Spray on Liners in Underground Coal Mine. Min. Technol..

[49] Xu Y., Wang L., Chu T., Liang D. (2014). Suspension Mechanism and Application of Sand-Suspended Slurry for Coalmine Fire Prevention. Int. J. Min. Sci. Technol..

[50] Xu Y., Wang D., Wang L., Zhong X., Chu T. (2012). Experimental Research on Inhibition Performances of the Sand-Suspended Colloid for Coal Spontaneous Combustion. Saf. Sci..

[51] Shi Q., Qin B., Bi Q., Qu B. (2018). Fly Ash Suspensions Stabilized by Hydroxypropyl Guar Gum and Xanthan Gum for Retarding Spontaneous Combustion of Coal. Combust. Sci. Technol..

[52] Ren X., Hu X., Xue D., Li Y., Shao Z., Dong H., Cheng W., Zhao Y., Xin L., Lu W. (2019). Novel Sodium Silicate/Polymer Composite Gels for the Prevention of Spontaneous Combustion of Coal. J. Hazard. Mater..

[53] Hu S., Xue S. (2011). Gel Fire Suppressants for Controlling Underground Heating. J. Coal Sci. Eng. (China).

[54] Huang Z., Sun C., Gao Y., Ji Y., Wang H., Zhang Y., Yang R. (2018). R&D of Colloid Components of Composite Material for Fire Prevention and Extinguishing and an Investigation of Its Performance. Process Saf. Environ. Prot..

[55] Cheng W., Hu X., Xie J., Zhao Y. (2017). An Intelligent Gel Designed to Control the Spontaneous Combustion of Coal: Fire Prevention and Extinguishing Properties. Fuel.

[56] Hu X., Cheng W., Shao Z. (2015). Novel Authigenic Gas Foaming Hydrogels for Preventing Coal Spontaneous Combustion. e-Polymers.

[57] Qin B., Dou G., Wang Y., Xin H., Ma L., Wang D. (2017). A Superabsorbent Hydrogel–Ascorbic Acid Composite Inhibitor for the Suppression of Coal Oxidation. Fuel.

[58] Zhang L., Qin B., Shi B., Wen K. (2016). Formation Mechanism of Foamed Gel for Controlling the Coal Spontaneous Combustion. Combust. Sci. Technol..

[59] Zhang L., Qin B. (2014). Study on the Gelation of Foamed Gel for Preventing the Spontaneous Combustion of Coal. J. Spectrosc..

[60] Zhang L., Qin B. (2016). Rheological Characteristics of Foamed Gel for Mine Fire Control. Fire Mater..

[61] Zhang L., Qin B., Shi B., Wu Q., Wang J. (2016). The Fire Extinguishing Performances of Foamed Gel in Coal Mine. Nat. Hazards.

[62] Shao Z., Wang D., Wang Y., Zhong X., Tang X., Hu X. (2015). Controlling Coal Fires Using the Three-Phase Foam and Water Mist Techniques in the Anjialing Open Pit Mine, China. Nat. Hazards.

[63] Qin B., Lu Y., Li Y., Wang D. (2014). Aqueous Three-Phase Foam Supported by Fly Ash for Coal Spontaneous Combustion Prevention and Control. Adv. Powder Technol..

[64] Zhang L., Qin B. (2014). Development of a New Material for Mine Fire Control. Combust. Sci. Technol..

[65] Lei S., Guo Q., Zhang D., Shi J., Liu L., Wei X. (2010). Preparation and Properties of the Phenolic Foams with Controllable Nanometer Pore Structure. J. Appl. Polym. Sci..

[66] Hu X., Zhao Y., Cheng W., Wang D., Nie W. (2014). Synthesis and Characterization of Phenol-Urea-Formaldehyde Foaming Resin Used to Block Air Leakage in Mining. Polym. Compos..

[67] Hu X., Wang D., Cheng W., Zhou G. (2014). Effect of Polyethylene Glycol on the Mechanical Property, Microstructure, Thermal Stability, and Flame Resistance of Phenol–Urea–Formaldehyde Foams. J. Mater. Sci..

[68] Hu X., Cheng W., Li C., Wang G., Lin X., Liu Z. (2016). Effects of Surfactants on the Mechanical Properties, Microstructure, and Flame Resistance of Phenol–Urea–Formaldehyde Foam. Polym. Bull..

[69] Hu X., Cheng W., Wang D. (2014). Properties and Applications of Novel Composite Foam for Blocking Air Leakage in Coal Mine. Russ. J. Appl. Chem..

[70] Wu J., Yan H., Wang J., Wu Y., Zhou C. (2013). Flame Retardant Polyurethane Elastomer Nanocomposite Applied to Coal Mines as Air-Leak Sealant. J. Appl. Polym. Sci..

[71] Hu X., Wang D., Wang S. (2013). Synergistic Effects of Expandable Graphite and Dimethyl Methylphosphonate on the Mechanical Properties, Fire Behavior, and Thermal Stability of a Polyisocyanurate-Polyurethane Foam. Int. J. Min. Sci. Technol..

[72] Song H., Liu J., Xue F., Cheng F. (2016). Flame Retardant Properties of Gas Sealing Materials Used in Coal Mines. Polimery.

[73] Twardowska I., Stefaniak S. (2006). Fly Ash as a Sealing Material for Spontaneous Combustion and Acid Rock Drainage Prevention and Control. Coal Combustion Byproducts and Environmental Issues.

[74] Su Y., Wang L., Zhang F. (2018). A Novel Process for Preparing Fireproofing Materials from Various Industrial Wastes. J. Environ. Manag..

[75] Weng T., Lin W., Li C. (2017). Properties Evaluation of Repair Mortars Containing EVA and VA/VeoVa Polymer Powders. Polym. Polym. Compos..

[76] Tosun A. (2017). Development of a Technology to Prevent Spontaneous Combustion of Coal in Underground Coal Mining. J. South. Afr. Inst. Min. Metall..

[77] Zhang R., Nie B., He X., Wang C., Zhao C., Dai L., Li Q., Liu X., Li H. (2011). Different Gas Explosion Mechanisms and Explosion Suppression Techniques. Procedia Eng..

[78] Chaunsali P., Mondal P. (2015). Influence of Mineral Admixtures on Early-Age Behavior of Calcium Sulfoaluminate Cement. ACI Mater. J..

[79] Chen X., Zhang Y., Sun W., Ge X., Zhao R. (2015). Research on Expansion Property of Inorganic Hole Sealing Material of Coal Mine. Mater. Res. Innov..

[80] Xiang X., Zhai C., Xu Y., Yu X., Xu J. (2015). A Flexible Gel Sealing Material and a Novel Active Sealing Method for Coal-Bed Methane Drainage Boreholes. J. Nat. Gas. Sci. Eng..

[81] Zhou A., Wang K. (2019). A New Inorganic Sealing Material Used for Gas Extraction Borehole. Inorg. Chem. Commun..

[82] Zhai C., Hao Z., Lin B. (2011). Research on a New Composite Sealing Material of Gas Drainage Borehole and Its Sealing Performance. Procedia Eng..

[83] Zhai C., Xiang X., Zou Q., Yu X., Xu Y. (2016). Influence Factors Analysis of a Flexible Gel Sealing Material for Coal-Bed Methane Drainage Boreholes. Environ. Earth Sci..

[84] Yang H., Zhang C., Li Z., Zhang J. (2016). Experimental Study on Sealing Material Characteristics for New Gas Extraction Boreholes. Saf. Coal Mines.

[85] Li B., Zhang J., Wei J., Zhang Q. (2018). Preparation and Sealing Performance of a New Coal Dust Polymer Composite Sealing Material. Adv. Mater. Sci. Eng..

[86] Zhang J., Li B., Sun Y. (2018). Performance of a New Coal-Dust-Based Composite Sealing Material for Gas-Drainage Borehole. Mater. Express.

[87] Ge Z., Mei X., Lu Y., Tang J., Xia B. (2015). Optimization and Application of Sealing Material and Sealing Length for Hydraulic Fracturing Borehole in Underground Coal Mines. Arab. J. Geosci..

[88] Trifunović P., Tokalić R., Ganić A. (2013). Application of Polymer Composites for Stabilization of Degraded Rock Mass in Mining. Undergr. Min. Eng..

[89] Murphy M.M. (2016). Shale Failure Mechanics and Intervention Measures in Underground Coal Mines: Results from 50 Years of Ground Control Safety Research. Rock Mech. Rock Eng..

[90] Lukey C., Spinks G., Baafi E., Porter I., Nemcik J. Polymer-Based Alternative to Steel Mesh for Coal Mine Strata Reinforcement. Proceedings of the Coal 2008: Coal Operators’ Conference.

[91] Nemcik J., Porter I., Baafi E., Lukey C. Geotechnical assessment of polymeric materials as skin reinforcement in underground mines. Proceedings of the Coal 2009: Coal Operators’ Conference.

[92] Nemcik J., Porter I., Baafi E., Towns J. Bearing Capacity of a Glass Fibre Reinforced Polymer Liner. Proceedings of the 11th Underground Coal Operators’ Conference.

[93] Nemcik J., Baafi E., Porters I. (2013). Stabilising rock surfaces with a glass reinforced polymer skin. Acta Geodyn. Geomater..

[94] Shan Z., Porter I., Nemcik J., Baafi E. (2019). Investigating the Behaviour of Fibre Reinforced Polymers and Steel Mesh when Supporting Coal Mine Roof Strata Subject to Buckling. Rock Mech. Rock Eng..

[95] Sun C., Wang X., Gao W. (2016). High Strength Polyester Flexible Web Application in Slice Mining. Shandong Coal Sci. Technol..

[96] Qi Z., Song H. (2003). Application and Prospect of Steel Fiber Reinforced Concrete in Underground Engineering. Concrete.

[97] Shahbazpanahi S., Abang A.A.A., Kamgar A., Farzadni N. (2015). Fracture Mechanic Modeling of Fiber Reinforced Polymer Shear-Strengthened Reinforced Concrete Beam. Compos. Part B.

[98] Xu J., Ma Z., Shi L., Wei C. (2013). Chemical Fiber Concrete Wet-Spraying Technology in Deep Rock Roadway. Saf. Coal Mines.

[99] Han Y., Jiang B., Yan J. (2009). Development of JL-1 Non-Alkaline Liquid Accelerator of Shotcrete. Materiai and Adminicle.

[100] Zhou G., Cheng W., Cao S. (2015). Development of a New Type of Alkali-Free Liquid Accelerator for Wet Shotcrete in Coal Mine and Its Engineering Application. Adv. Mater. Sci. Eng..

[101] Li X., Hao J. (2018). Orthogonal Test Design for Optimization of Synthesis of Super Early Strength Anchoring Material. Constr. Build. Mater..

[102] Spearing A.J.S., Greer B., Reilly M. (2011). Improving Rockbolt Installations in US Coal Mines. J. South. Afr. Inst. Min. Metall..

[103] Han J., Xie W., Zhang M., Zhang H., Cao C. (2017). Experimental Study on the Effect of Resin Anchoring Agent Mixing with Steel Grit. Electron. J. Struct. Eng..

[104] Su X., Du X., Yuan H., Li B. (2016). Research of the Thermal Stability of Structure of Resin Anchoring Material Based on 3D CT. Int. J. Adhes. Adhes..

[105] Wang Z. (2016). Development and Application of New Type Waterproof Anchorage Agent. Coal Engineeing.

[106] Contrafatto L., Cosenz R. (2014). Behaviour of Post-Installed Adhesive Anchors in Natural Stone. Constr. Build. Mater..

[107] Kang H. (2014). Support Technologies for Deep and Complex Roadways in Underground Coal Mines: A Review. Int. J. Coal Sci. Technol..

[108] Qin B., Lu Y., Li F., Jia Y., Zhu C., Shi Q. (2014). Preparation and Stability of Inorganic Solidified Foam for Preventing Coal Fires. Adv. Mater. Sci. Eng..

[109] Lu Y., Qin B. (2015). Mechanical Properties of Inorganic Solidified Foam for Mining Rock Fracture Filling. Mater. Express.

[110] Hu X., Zhao Y., Cheng W. (2015). Effect of Formaldehyde/Phenol Ratio (F/P) on the Properties of Phenolic Resins and Foams Synthesized at Room Temperature. Polym. Compos..

[111] Zhang J., Sun Y. (2019). Experimental and Mechanism Study of a Polymer Foaming Grouting Material for Reinforcing Broken Coal Mass. KSCE. J. Civ. Eng..

[112] Guan X., Yang Z., Zhang C. (2012). Bonding Mechanism between Waterglass/Polyurethane Adhesive and Coal. J. Adhes..

[113] He Z., Li Q., Wang J., Yin N., Jiang S., Kang M. (2016). Effect of Silane Treatment on The Mechanical Properties of Polyurethane/Water Glass Grouting Materials. Constr. Build. Mater..

[114] Zhang Q., Hu X., Wu M., Zhao Y., Yu C. (2018). Effects of Different Catalysts on the Structure and Properties of Polyurethane/Water Glass Grouting Materials. J. Appl. Polym. Sci..

[115] Gao X., Wang X., Liu X. (2018). New Chemical Grouting Materials and Rapid Construction Technology for Inclined Shaft Penetrating Drift-Sand Layer in Coal Mine. Adv. Mater. Sci. Eng..

